# Luminescent platinum(II) complexes with functionalized N-heterocyclic carbene or diphosphine selectively probe mismatched and abasic DNA

**DOI:** 10.1038/ncomms10655

**Published:** 2016-02-17

**Authors:** Sin Ki Fung, Taotao Zou, Bei Cao, Tianfeng Chen, Wai-Pong To, Chen Yang, Chun-Nam Lok, Chi-Ming Che

**Affiliations:** 1 State Key Laboratory of Synthetic Chemistry, Institute of Molecular Functional Materials, Chemical Biology Centre, and Department of Chemistry, The University of Hong Kong, Pokfulam Road, Hong Kong, China; 2 HKU Shenzhen Institute of Research and Innovation Shenzhen, Shenzhen 518053, China; 3 Department of Chemistry, Jinan University, Guangzhou 510632, China

## Abstract

The selective targeting of mismatched DNA overexpressed in cancer cells is an appealing strategy in designing cancer diagnosis and therapy protocols. Few luminescent probes that specifically detect intracellular mismatched DNA have been reported. Here we used Pt(II) complexes with luminescence sensitive to subtle changes in the local environment and report several Pt(II) complexes that selectively bind to and identify DNA mismatches. We evaluated the complexes' DNA-binding characteristics by ultraviolet/visible absorption titration, isothermal titration calorimetry, nuclear magnetic resonance and quantum mechanics/molecular mechanics calculations. These Pt(II) complexes show up to 15-fold higher emission intensities upon binding to mismatched DNA over matched DNA and can be utilized for both detecting DNA abasic sites and identifying cancer cells and human tissue samples with different levels of mismatch repair. Our work highlights the potential of luminescent Pt(II) complexes to differentiate between normal cells and cancer cells which generally possess more aberrant DNA structures.

## Abstract (web summary)

DNA pairing defects such as mismatched and abasic DNA are prevalent in cancer cells. Here, the authors present luminescent platinum based probes capable of preferentially binding to mismatched and abasic DNA, and reporting this by a significant luminescence enhancement

## Graphical abstract

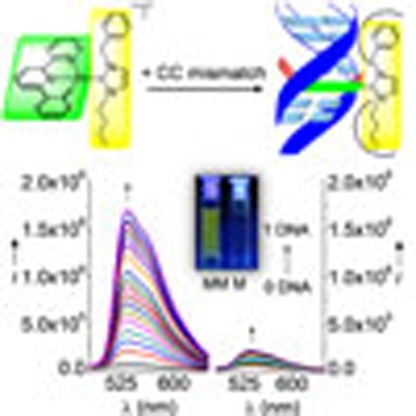

The binding interactions between metal complexes and nucleic acids have been the subject of numerous studies in recent decades^1^,^2^,^3^,^4^. These interactions are likely to lead to therapeutic (for example, anti-cancer) effects and/or be used for diagnostic (for example, luminescent probes) purposes^5^,^6^,^7^,^8^,^9^,^10^,^11^. To date, the selective targeting of nucleic acids in cancer cells by chemotherapeutic metal complexes in a manner that minimizes off-target bindings and hence diminishes side effects remains a great challenge^12^,^13^. Endeavours in this research area have led to luminescent metal complexes that show emission responses with specificity for specific nucleic acid structures, such as G-quadruplex DNA^14^,^15^, double-stranded RNA^16^,^17^ and mismatched DNA^13^,^18^,^19^. The recognition of mismatched DNA is of importance for cancer diagnosis and therapy, because DNA mismatches are associated with oncogenic transformation^20^,^21^,^22^. Furthermore, cancer cells, such as those of colorectal cancer, show a high frequency of DNA mismatches as a result of their deficiency in mismatch repair (MMR)^13^,^23^,^24^,^25^,^26^. Pioneering work by Barton and co-workers has shown that the octahedral d^6^ metal complexes [Rh(bpy)_2_chrysi]^3+^ (bpy=2-phenylpyridine, chrysi=5,6-chrysenequinone diimine)^18^,^27^,^28^ and Δ-[Ru(bpy)_2_dppz]^2+^ (dppz=dipyrido[3,2-*a*:2′,3′-c]phenazine)^19^,^29^,^30^ are able to selectively bind to mismatched DNA. Other researchers have reported that octahedral cobalt(III) complexes^31^, a six-coordinated silicon complex containing a sandwich ruthenium moiety^32^, some non-metal compounds^33^,^34^,^35^,^36^,^37^,^38^,^39^,^40^,^41^ and others^13^ can recognize DNA mismatches with specificity.

The d^8^ pincer Pt(II) complexes have been well documented to intercalate into matched DNA^1^,^2^,^4^,^11^,^42^. With intrinsic emission properties that are highly sensitive to the microenvironment, these types of Pt(II) complexes have been developed as luminescent DNA probes and have been identified as potent therapeutic agents for cancer treatment^14^,^17^,^43^,^44^,^45^. However, metal complexes with bare planar coordination geometries likely exhibit non-discriminative intercalative binding interactions with DNA. In the crystal structures of mismatched DNA bound to Rh(III)^18^,^28^ or Ru(II)^19^ complexes, the planar extended π-conjugated ligands (with a width of up to 11.3 Å and a length of up to 9.4 Å) favour strong π-stacking interactions with nearby base pairs at mismatched sites, concomitantly with the ejection of mismatched base pairs, whereas the other auxiliary bipyridine ligands promote a groove binding interaction. We therefore hypothesized that a pincer Pt(II) complex coordinated with an out-of-plane bulky ancillary ligand may exhibit selective binding affinity towards mismatched DNA, as discussed in the next section. The bulky ancillary ligand can be designed to hamper the intercalation of the platinum complex with well-matched DNA while exhibiting high binding affinity with mismatched DNA. This difference is possible because the mismatched sites possess larger binding pockets. We have previously described a luminescent [Pt^II^(ĈN̂N)(NHC^2C4^)]^+^ (HĈN̂N=6-phenyl-2,2′-bipyridine, NHC=*N*-heterocyclic carbene, **1b** in the current report) complex with an NHC ligand almost perpendicular to the C-deprotonated ĈN̂N plane^46^. This complex exhibits a weak binding affinity to matched DNA. Thus, varying the *N*-alkyl/aryl substitution(s) of NHC, [Pt^II^(ĈN̂N)(NHC)]^+^ may generate a candidate scaffold to target mismatched DNA via cooperative π-stacking and groove binding interactions.

Here we describe several luminescent, mismatched DNA probes based on the [Pt^II^(ĈN̂N)(NHC)]^+^, [Pt^II^(N̂ĈN)(NHC)]^+^ and [Pt^II^_2_(ĈN̂N)_2_(μ-dcpm)]^2+^ complexes. The mononuclear Pt(II) complex selectively detects DNA containing CC mismatches, and the dinuclear Pt(II) complex exhibits high selectivity towards several types of DNA mismatches. These luminescent Pt(II) complexes also selectively bind thermodynamically unstable abasic DNA, are able to differentiate cancer cells that have different levels of MMR capacity and can differentiate colon tumour tissue from normal tissue.

## Results

### Structure and emission responses to CC mismatched DNA

**Figure 1:**
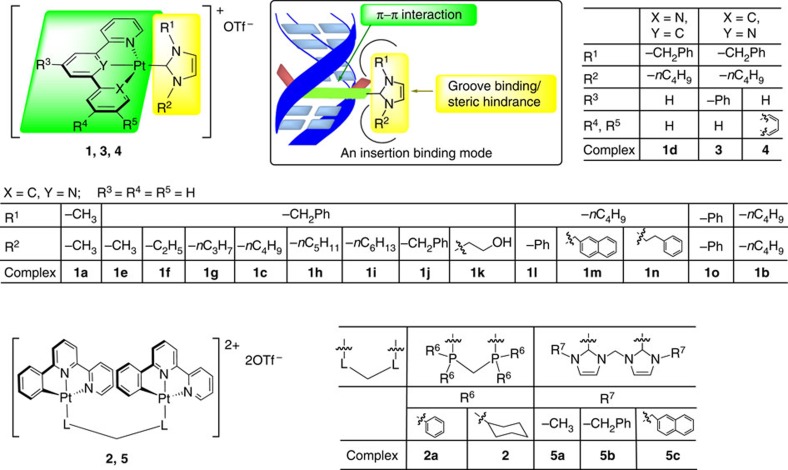
Chemical structures and proposed binding modes. Platinum(II) complexes **1a**–**1o**, **2**, **2a**, **3**, **4** and **5a**–**5c**. The counteranions for all complexes are CF_3_SO_3_^−^. The inset shows the molecular design of Pt(II) complexes tested for binding mismatched DNA.

**Figure 2:**
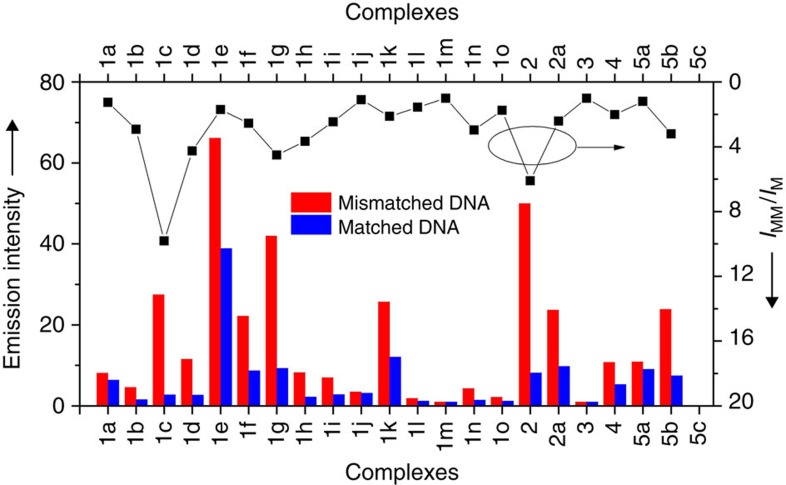
Emission responses of Pt(II) complexes to matched and mismatched DNA. A summary of emission intensities (at near level off) of different Pt(II) complexes towards mismatched and matched DNA and the emission intensity ratios (*I*_MM_/*I*_M_).

**Figure 3:**
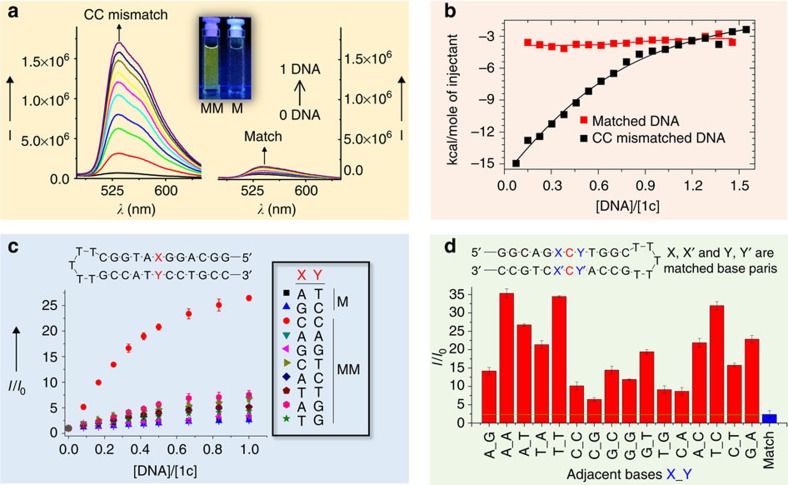
Binding of **1c** with mismatched DNA. (**a**) The emission spectra of complex **1c** (5 μM) in a Tris buffer solution (50 mM NaCl and 2 mM Tris, pH 7.5) after binding to different concentrations of CC mismatched DNA (MM) and matched DNA (M). (**b**) Plot of integrated ITC data for the exothermic interaction between CC mismatched DNA (0.75 mM) and **1c** (0.1 mM). (**c**) Changes in emission intensity (±s.e.m.) at 535 nm of complex **1c** (5 μM) in the Tris buffer solution upon the addition of different types of DNA. M, match; MM, mismatch. (**d**) Relative emission enhancement of **1c** towards CC MM DNA with different adjacent base pairs.

We synthesized and characterized a series of [Pt^II^(ĈN̂N)(NHC)]^+^ complexes with different alkyl chains, aromatic groups and hydrophilic alcohol moieties on NHC and/or on C-deprotonated ĈN̂N ligands (Fig. 1; details in Supplementary Information). In addition, we examined the emission responses of complexes that bind mismatched and matched DNA (Supplementary Fig. 1). The most unstable CC mismatched DNA was chosen in the primary screen because metalloinsertors display the highest binding to this type of mismatched DNA^29^. We found that as the steric hindrance of the NHC ligand increased, the binding of the Pt(II) complexes to matched DNA decreased, whereas the binding with CC mismatched DNA remained high for certain bulky NHC ligands (Fig. 2; the DNA sequences in all the experiments are depicted in the Supplementary Table 2). For example, for the [Pt(ĈN̂N)(NHC)]^+^ type complexes of **1e** (−CH_3_), **1f** (−C_2_H_5_), **1g** (−C_3_H_7_), **1c** (−C_4_H_9_), **1h** (−C_5_H_11_), **1i** (−C_6_H_13_) and **1j** (−CH_2_Ph), which contain the same *N*^3^-benzyl group but different *N*^1^-substituents on the NHC ligands (Table 1), an increase in the length of the *N*^1^-alkyl was accompanied by a marked decrease in emission enhancement towards matched DNA (*I*_M_), from 38.9-fold to <3-fold. Although increasing the chain length from the *N*^1^-methyl of **1e** to the *N*^1^-ethyl of **1f** led to a decrease in emission enhancement in the case of mismatched DNA (*I*_MM_), the emission intensity of **1g**, which bears *N*^1^-propyl, was much stronger than that of **1f**, suggesting that *N*^1^-propyl favours the groove binding of the Pt(II) complex to DNA. This may enhance the tightness of the insertion binding mode, as proposed. Further lengthening the alkyl chain to *n*-butyl, *n*-pentyl and *n*-hexyl gradually decreased the binding with mismatched DNA. Comparing the *I*_MM_/*I*_M_ ratios, **1c**, containing *N*^1^-(*n*-butyl) and *N*^3^-benzyl substitutions on the NHC (Fig. 1) showed the most significantly different emission responses between CC mismatched DNA and matched DNA. We characterized the structure of this complex by ^1^H–^1^H correlation spectroscopy (COSY) and nuclear Overhauser effect spectroscopy (NOESY) nuclear magnetic resonance (NMR; Supplementary Fig. 2). As shown in Fig. 3a,c, the emission intensity of **1c** (*λ*_max_=535 nm) in a Tris-buffered solution increased by 26.4-fold in the presence of 1 equivalent of a hairpin DNA oligomer with a CC mismatch^29^ (Fig. 3c). However, the emission of **1c** was enhanced only 2.6-fold upon the addition of the same amount of the hairpin DNA, having the cognate sequence with the mismatched bases replaced by matched GC pairs. In another mismatched DNA model using a 17-mer double-stranded DNA (dsDNA) formed by two complementary oligomers^47^, the emission intensity of **1c** displayed 27.5-fold and 2.8-fold increases in emission intensity upon the addition of CC mismatched 17-mer dsDNA and matched 17-mer dsDNA, respectively (Supplementary Fig. 3). In the literature, a rhodium-Oregon Green conjugate has been reported to exhibit a ∼3.2-fold higher emission intensity in the presence of CC mismatched DNA compared with matched DNA^47^. In a control experiment, the classical DNA intercalator, ethidium bromide (EB), showed increases in emission intensity with hairpin mismatched DNA and matched DNA of 12.7-fold and 14.1-fold, respectively, revealing a 0.9-fold difference (Supplementary Fig. 4). Under similar conditions, **1a**, containing a less bulky *N*-methyl substitution on the NHC, displayed similar 8.5-fold and 7.1-fold emission enhancements for hairpin CC mismatched DNA and matched DNA, respectively (1.2-fold difference; Supplementary Fig. 5). This result can be explained by the fact that the less bulky *N*-methyl substituted NHC does not impose enough steric hindrance to diminish the binding of the Pt complex with matched DNA, resulting in similarly high enhancement in emission intensity for both matched and mismatched DNA. Conversely, the more bulky *N*^1^-(*n*-butyl) and *N*^3^-benzyl groups diminished the binding of **1c** with matched DNA, while a much stronger binding affinity with mismatched DNA was still maintained.

### Characterizations of bindings to CC mismatched DNA

**Figure 4:**
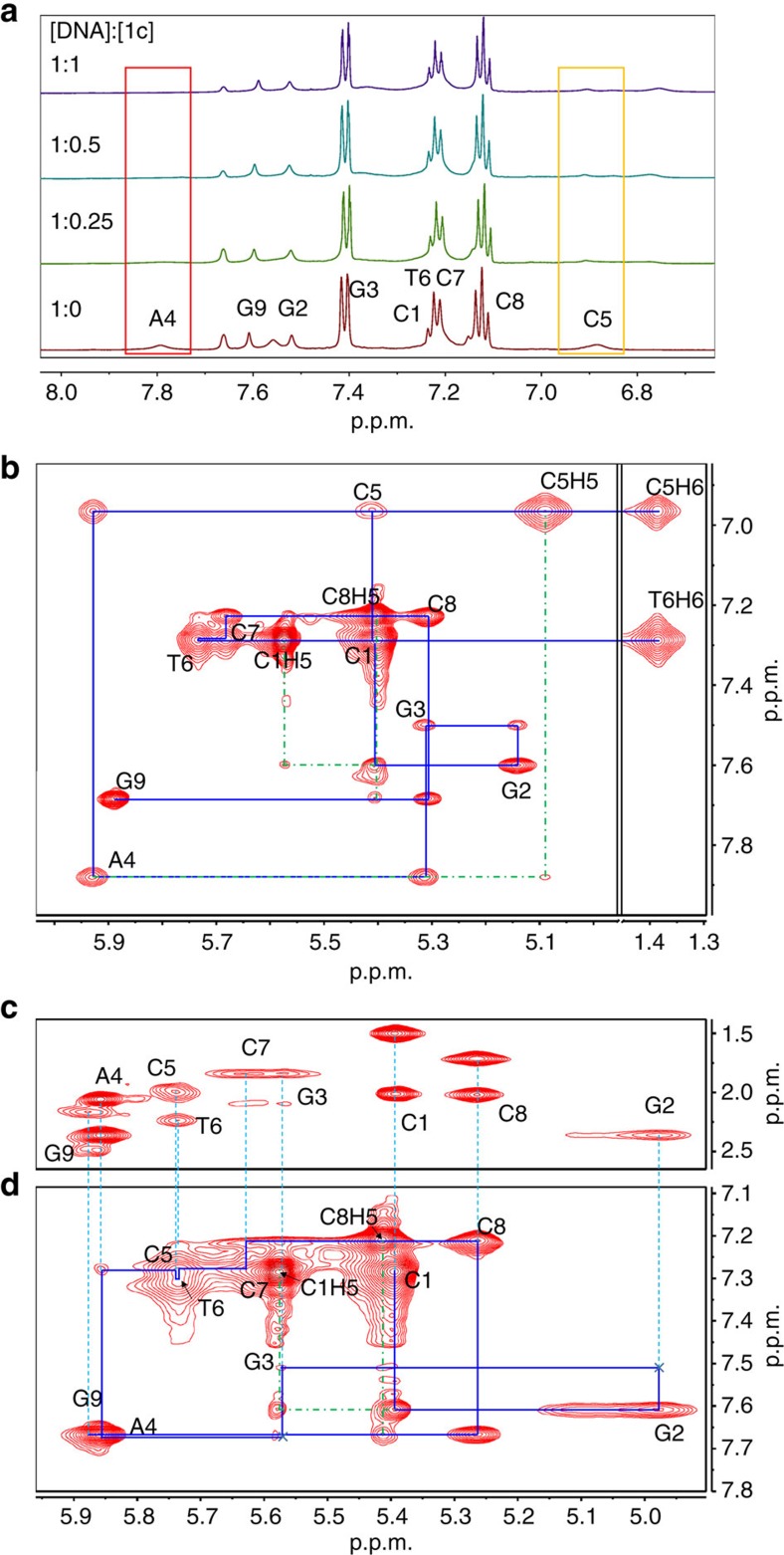
NMR analysis of the binding of 1c with mismatched DNA. (**a**) ^1^H NMR (phosphate buffer=50 mM, pH 6.1, NaCl=20 mM, in D_2_O, 10 °C) spectra of a DNA oligonucleotide in the presence of different amounts of **1c**. (**b**) 2D H1′ × aromatic (left) and T(Me) × aromatic (right) NOESY spectra of a DNA oligonucleotide in phosphate buffer (50 mM in D_2_O, containing NaCl=20 mM, 10 °C); a sequential NOESY walk along the full strand was identified. (**c**) TOCSY spectrum of H1′ × H2′–H2′′ and (**d**) NOESY spectrum of H1′ × aromatic of a DNA oligonucleotide in the presence of an equimolar concentration of **1c** in phosphate buffer (50 mM in D_2_O, containing NaCl=20 mM, 10 °C). We also found a sequential NOESY walk that was significantly different at A4 and C5 in comparison with DNA only in **b**, suggestive of interactions of **1c** at a mismatched site. Note that the interactions of C1H5 with G2H8 and C8H5 with G9H8 at the terminal sites in **b** and **d**, and the interaction of C5H5 with A4H8 at the mismatched site in **b** were identified.

The following experiments were performed to shed light on the DNA-binding interactions of the novel complexes. In ultraviolet/visible absorption titration experiments, upon the addition of dsDNA containing a CC mismatch to **1c** in a Tris-buffered solution, a hypochromicity of ∼34% at *λ*=335 nm, together with isosbestic spectral changes, was observed (Supplementary Fig. 6a); however, only a minor hypochromicity of ∼12% was detected at the same wavelength for **1c** when matched dsDNA with a similar sequence was used instead (Supplementary Fig. 6b). Isothermal titration calorimetry (ITC) experiments (Fig. 3b) revealed that the titration of CC mismatched DNA with **1c** resulted in a significant exothermic reaction, revealing binding between the two components, with a dissociation constant (*K*_d_) of 51.5±8.0 μM. In contrast, the reaction between **1c** and matched DNA was a very weak exothermic reaction, with a *K*_d_ estimated to be >600 μM. In a similar experiment, the *K*_d_ values of the binding of **1a** to mismatched and matched DNA were 66.2±15.7 and 56.4±13.6 μM, respectively (Supplementary Fig. 7a,b). The *K*_d_ values of the binding of EB to mismatched DNA and matched DNA were 1.32±0.24 and 1.60±0.28 μM, respectively (Supplementary Fig. 8a,b). ^1^H NMR and two-dimensional (2D) NOESY, total correlation spectroscopy (TOCSY) and COSY NMR experiments were performed using a self-complementary DNA oligonucleotide, 5′-C_1_G_2_G_3_A_4_*C*_5_T_6_C_7_C_8_G_9_-3′, containing CC mismatches in D_2_O (phosphate buffer, pH 6.1; Fig. 4a–d; Supplementary Figs 9 and 10)^48^. In the range of 6.8–8.0 p.p.m., the aromatic ^1^H signals of C5 and A4 show substantial shifts, whereas those of the other bases remain nearly unshifted in the presence of **1c** (^1^H NMR in Fig. 4a). In addition, in the 2D H1′ × aromatic NOESY spectrum of **1c** with DNA at a 1:1 ratio, a sequential NOESY walk (Fig. 4d), which showed a marked shift at A4 and C5 compared with DNA only (Fig. 4b), was found in combination with cytosine H6/H5 TOCSY (Supplementary Fig. 9) and H1′ × H2′–H2′′ TOCSY (Fig. 4c) NMR. At [**1c**]:[DNA] ratios of 0.25:1 and 0.5:1 (Supplementary Fig. 10), a gradual disappearance of C5 and A4 aromatic protons of complex-free DNA and the subsequent appearance of new C5 and A4 aromatic protons of **1c**-bound DNA in the NOESY spectra were found. Thus, both one-dimensional and 2D NMR results indicate the possible binding of **1c** at the mismatched site. Collectively, the more significant the hypochromicity in ultraviolet/visible spectral changes, the higher the amount of heat generation, and the marked shifts of NMR signals at the mismatched site together revealed that **1c** preferentially binds to DNA containing CC mismatches. In the emission quenching experiments using [Cu(phen)_2_]^2+^ (phen=1,10-phenanthroline), a minor-groove-specific quencher, the emission intensity of **1c** in the presence of CC mismatched DNA gradually decreased upon increasing the amount of [Cu(phen)_2_]^2+^ (Supplementary Fig. 11), indicating the binding of the complex through the minor groove^29^. Indeed, a favourable binding mode between **1c** and CC mismatched DNA has also been identified in molecular docking experiments (Supplementary Fig. 12), consistently with the insertion of the Pt(ĈN̂N) plane between base pairs and the favourable minor-groove binding interaction of the *N*^1^-(*n*-butyl) and *N*^3^-benzyl moieties. Complex **1d**, which contains an N̂ĈN instead of a ĈN̂N ligand (Fig. 1), exhibited 4.5-fold higher emission responses towards 1 equivalent of CC mismatched DNA than matched DNA (Supplementary Fig. 13).

The emission responses of **1c** towards all other types of DNA mismatches, including CT, AC, TT, AG, TG, AA and GG mismatches, all of which are more thermodynamically stable than CC mismatches^49^, were tested (Fig. 3c). However, no significant difference in emission responses between these mismatched DNA and matched DNA was observed. We also examined the emission responses of **1c** to CC mismatched DNA having 16 different adjacent base pairs (Fig. 3d). Complex **1c** displayed higher emission responses to all the CC mismatched DNA sequences than the cognate matched DNA, with adjacent 5′-A*C*A-3′ revealing the highest emission enhancement (15.0-fold higher than matched DNA). These experiments suggest that **1c** is a selective probe for CC mismatched DNA.

### Targeting thermodynamically more stable DNA mismatches

**Figure 5:**
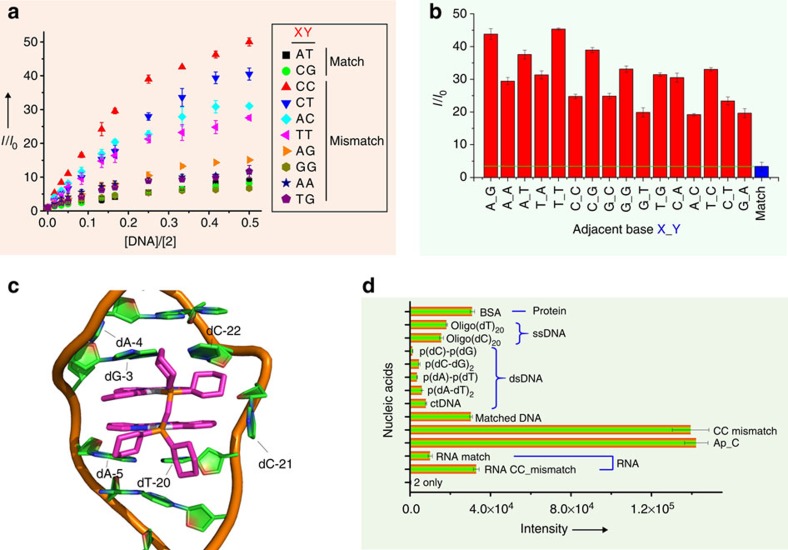
Binding of 2 with mismatched DNA. (**a**) Changes in emission intensity (±s.e.m.) at 634 nm of **2** (5 μM) in Tris buffer solution (50 mM NaCl, 2 mM Tris, pH 7.5) after the addition of different concentrations of various DNA. (**b**) Relative emission enhancement of **2** towards CC mismatched DNA with different adjacent base pairs. (**c**) QM/MM optimized structure of mismatched DNA bound to **2**. (**d**) Emission responses (intensity±s.e.m.) of **2** towards different analytes.

For recognition of the more stable DNA mismatches, an increase in the binding affinity of the Pt(II) complex with DNA is required. The dinuclear analogues of **1c**, which contain an additional positive charge and a larger molecular dimension, are expected to have higher DNA-binding affinities as a result of stronger ionic bonding interactions and possibly a better geometry to fit into the pocket at the mismatched site. Therefore, we examined the emission responses of a series of doubly charged dinuclear platinum complexes with two Pt^II^(ĈN̂N) moieties linked by bridging bis(NHC) or diphosphine ligands (Fig. 1) in the presence of hairpin mismatched and matched DNA. Among the dinuclear Pt(II) complexes, [Pt^II^_2_(ĈN̂N)_2_(μ-dcpm)]^2+^ **2** (Fig. 1; the X-ray crystallographic structure is depicted in Supplementary Fig. 14 and described in Supplementary Table 1, showing an intramolecular Pt–Pt distance of 3.201 Å) was observed to display higher selectivity towards hairpin CC mismatched DNA (Fig. 2). Figure 5a and Supplementary Fig. 15 illustrate that the emission intensity of **2** at *λ*=634 nm was elevated 50-fold upon the addition of only 0.5 equivalents of hairpin DNA containing a CC mismatch but enhanced only 8.2-fold in the presence of the same amount of matched DNA, that is, a 6.1-fold difference in emission responses.

The binding affinity of **2** with DNA was examined by ITC. The interaction of **2** was much more exothermic with CC mismatched DNA than with matched DNA (Supplementary Fig. 16). Notably, the *K*_d_ of the binding of **2** to CC mismatched DNA was found to be 7.0±2.1 μM, which is 7.4-fold higher than that of **1c**. The *K*_d_ of the binding of **2** with matched DNA was negligible. In addition, the melting temperature (*T*_m_), as determined by the change in absorbance (OD_260nm_) of DNA was increased by 6.8 °C for mismatched DNA in the presence of **2** at an equimolar concentration (Supplementary Fig. 17a). However, only a 0.5 °C increase in melting temperature was found for matched DNA in the presence of **2** (Supplementary Fig. 17b).

Complex **2** also exhibited higher emission responses to CC mismatched DNA containing 16 different adjacent sequences (Fig. 5b), with a nearby 5′-T*C*T-3′ sequence generating the highest fold increase in emission (13.5-fold higher than matched DNA). In addition to its response to CC mismatches, **2** also showed higher emission responses to several other types of mismatched DNA over matched DNA, and the emission increased 4.9-fold for CT, 3.8-fold for AC and 3.4-fold for TT compared with matched DNA but no higher for other mismatches (AG, 1.8-fold; GT, 1.4-fold; AA, 1.4-fold; GG, 0.8-fold; Fig. 5a). Such differences in the emission responses between mismatched and matched DNA partially correlated with the thermodynamic stability of the DNA base pairs, CC≤AC≤CT<AA≤TT<GA≤GT<GG^49^, suggesting an insertion binding mode^50^. The emission response to AC was slightly lower than that to CT, and the response to AA was lower than that to TT; these observations are not fully consistent with the trend of thermodynamic stability of mismatched DNA. The lower emission response towards A-containing AC and AA mismatches may have been caused by the weak coordination of the N7 of purine in adenine at the mismatch site to the Pt complex, leading to decrease of emission intensity (note that the pyrimidine N3 in C and T is not accessible to the Pt(II) complexes due to steric hindrance). We performed hybrid quantum mechanics/molecular mechanics (QM/MM) calculations to study the mode of binding between **2** and mismatched DNA. As shown in Fig. 5c, **2** bound to mismatched DNA through an insertion binding mode at the minor groove (this result is also consistent with the results of a [Cu(phen)_2_]^2+^ quenching experiment, Supplementary Fig. 18); the ĈN̂N planes interacted with nearby GC and AT base pairs by π-stacking interactions (inter-planar distance between the ĈN̂N plane and the base pairs of ∼3.2 Å). The QM/MM simulation also indicated that the dinuclear structure better fit the mismatched sites, which have larger pockets (the inter-planar separation between nearby base pairs at mismatched sites can be >9 Å), but not the matched site, which contains less space between base pairs.

We also tested the emission responses of **2** to dsDNA of poly(dC)-poly(dG), poly(dC-dG)_2_, poly(dA)-poly(dT) and poly(dA-dT)_2_; single-stranded DNA of oligo(dT)_20_ and oligo(dC)_20_; dsRNA and bovine serum albumin, all of which were observed to have at least 4.5-fold lower emission intensity than that of CC mismatched DNA (Fig. 5d). Notably, the emission intensity of **2** with RNA containing a CC mismatch was 3.3-fold higher than that with matched RNA (Fig. 5d).

### Photophysical properties upon binding to mismatched DNA

To better understand the increase in emission upon binding with mismatched DNA, we measured the emission spectra of the Pt(II) complex (**1c** or **2**) in buffer solutions containing CC mismatched DNA under air or argon. Under Ar, no significant changes in emission intensity were found compared with that under air conditions (Supplementary Fig. 19). The two Pt(II) complexes were weakly emissive in buffer solutions, but their emission intensities were significantly higher in acetonitrile, even under air (11-fold for **1c**, 9-fold for **2**, Supplementary Fig. 20a,b; the emission intensity was found to increase with decreasing donor strength of various solvents, as shown in Supplementary Fig. 20c). Thus, the increased emission could not be accounted for by the shielding of oxygen quenching upon mismatched DNA binding to the Pt(II) complexes. In addition, variations of pH from 5 to 11 did not evoke a significant change in emission intensity (Supplementary Fig. 21). We propose that the shielding of quenching by a buffer solution and/or a more ordered structure of the Pt(II) complex after mismatched DNA binding could account for the emission enhancement^51^,^52^.

We used time-resolved emission spectroscopy to examine the binding between the Pt complexes and DNA. As shown in Supplementary Fig. 22, the emission of **2** in the presence of CC mismatches had a lifetime of 2.3 μs, whereas in the presence of matched DNA, the lifetime is composed of a major portion of short decay (<100 ns) and a small amount of 1.2-μs decay. The difference in emission intensity, *I*_mismatch_/*I*_match_, was observed to increase with increasing decay time, from 2.3-fold at *t*=0 to 11.8-fold at *t*=10 μs (Supplementary Fig. 23). Therefore, time-resolved emission spectroscopy may be a useful strategy to achieve higher sensitivity than steady-state emission spectroscopy for the detection of mismatched DNA using luminescent Pt(II) complexes.

### Targeting abasic DNA

The abasic (AP) site, in which a purine or a pyrimidine base is missing in a nucleotide, represents another type of thermodynamically unstable DNA defect^50^. In the presence of a 27-mer dsDNA oligomer containing an abasic site^50^ (AP_C with a complementary base of C), the emission intensity of an equimolar amount of **1c** at *λ*_max_=535 nm exhibited a 32.2-fold increase (Supplementary Fig. 24). This elevation in emission was even higher than that observed for the binding of **1c** with CC mismatched DNA with similar sequences (26.4-fold). For comparison, only a 4.7-fold increase in emission was detected when **1c** was incubated with normal 27-mer dsDNA with a cognate sequence. In the presence of other abasic sites of AP_A, AP_T and AP_G, the emission intensities of **1c** were increased by 14.8, 21.1 and 14.4 times, respectively. Complex **2** showed 17.0–29.3-fold increases in emission intensity in the presence of different abasic DNA sequences, but only a 6.1-fold increase in emission intensity with normal DNA (Supplementary Fig. 25). We also measured the effects of **2** on the *T*_m_ of DNA bearing an abasic site (Supplementary Fig. 26). The melting temperature of abasic DNA in the presence of **2** was increased by 5.3 °C, whereas the *T*_m_ of the similar DNA sequence without an abasic site was not affected, indicating a high selectivity of **2** towards abasic DNA over matched DNA. Thus, these Pt(II) complexes could be used to probe DNA abasic sites. Such a property is also found in Rh(III)-based metalloinsertors, in which the metal complex binds to thermodynamically unstable DNA structures through an insertion binding mode^50^.

### Detection of genomic DNA base pair defects

**Figure 6:**
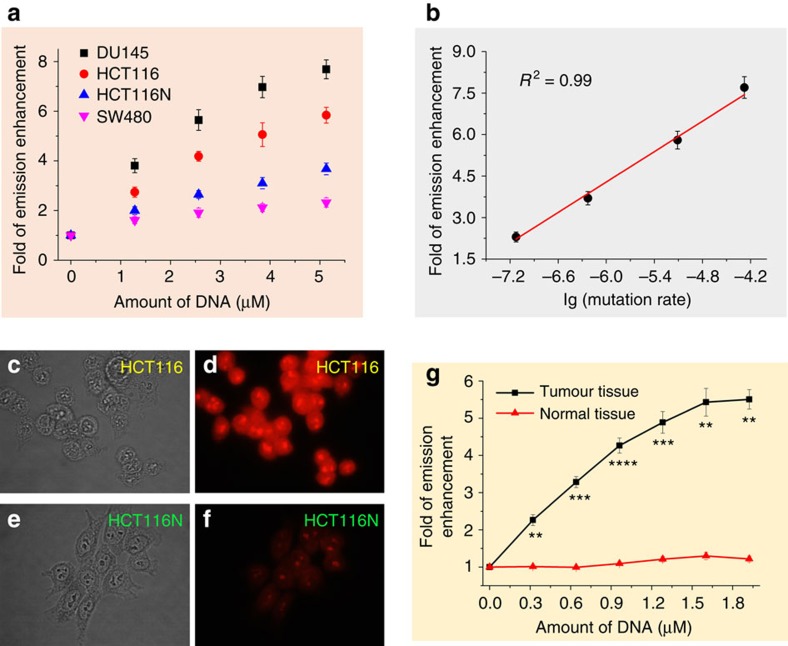
Detection of mismatched DNA in cancer cells. (**a**) Changes in emission intensity (±s.e.m.) of **2** (5 μM) at 634 nm in an aqueous buffer solution (50 mM NaCl and 2 mM Tris, pH 7.5) after binding to genomic DNA extracted from different cancer cell lines. (**b**) Plot of fold of increased emission versus reported mutation rates in different cancer cell lines, lg (mutation rate); a linear fit is shown. Fluorescence microscopy studies of HCT116 (**d**) and HCT116N (**f**) cells pretreated with digitonin and stained with 10 μM **2**; (**c**,**e**) bright-field images of HCT116 and HCT116N cells, respectively. (**g**) Plot of emission responses (fold of increase in emission±s.e.m.) of **2** versus different concentrations of DNA extracted from colon tumour tissue or adjacent normal tissue. All the concentrations of genomic DNA are shown as base pair concentrations.

The human colorectal carcinoma cell line HCT116 is known to be MMR deficient, owing to mutations in the MMR-associated *hMLH1* gene, whereas HCT116 cells containing a transferred, normal chromosome 3 (denoted as HCT116N) restore MMR activity^53^. We found that **2** showed significantly stronger emission at *λ*_max_=634 nm in the presence of genomic DNA isolated from HCT116 cells compared with that from HCT116N cells (*P*<0.01, *n*=3; Fig. 6a; Supplementary Fig. 27). We then measured and compared the emission responses of **2** with several types of cancer cells with different mutation rates. The human cancer cell lines DU145, HCT116, HCT116N and SW480 have mutation rates (mutations per cell per generation) in the *hprt* gene locus of 529 × 10^−7^, 77.5 × 10^−7^, 5.9 × 10^−7^ and 0.75 × 10^−7^, respectively^54^, and the relative emission intensities of **2** (5 μM) at 5.1 μM of DNA are 7.7, 5.8, 3.7 and 2.3, respectively (Fig. 6a shows an increase of emission intensity at 0–5.1 μM of genomic DNA from different cancer cells). Notably, the plot of the fold of emission increase versus the lg (mutation rate) yields a linear fit with *R*^2^=0.99, as shown in Fig. 6b, indicating a strong correlation between emission response and mutation rate.

We next tested whether **2** could be used to selectively detect mismatched DNA in permeabilized cell samples by fluorescence microscopy. Because the Pt(II) complexes are lipophilic and tend to accumulate in cytoplasmic compartments, the HCT116 and HCT116N cancer cells were permeabilized by digitonin and then washed to minimize the fluorescence background due to interactions of the Pt(II) complexes with cytoplasmic contents. As shown in Fig. 6c–f, after labelling with **2**, the emission signal of the HCT116 cells was significantly stronger than that of the HCT116N cells. In addition, complex **2** emitted significantly stronger signals in cancerous HCT116 cells compared with those in the immortalized normal human colon mucosal epithelial cell line NCM460 (ref. 55; Supplementary Fig. 28); complex **2** also consistently exhibited significantly lower emission responses in the presence of the DNA isolated from NCM460 cells compared with that from HCT116 cells (Supplementary Fig. 27). The differences in emission intensity were not caused by variations in cellular uptake of Pt(II) complexes as shown by inductively coupled plasma mass spectrometer measurements (Supplementary Fig. 29). For comparison, no significant differences in the emission properties were found between the two cell lines labelled with the general DNA intercalator EB (Supplementary Fig. 30).

We further examined the ability of the Pt(II) complex to detect mismatched DNA of primary human tumours of colon cancer which is frequently characterized by MMR deficiency. DNA samples were extracted from colorectal adenocarcinoma (well to moderately differentiated) and the surrounding normal tissues of a patient (see details in the Methods section). The results of the emission responses of **2** (5 μM) to the DNA from the tumour and normal tissues are shown in Fig. 6g. In the presence of 0.3–1.9 μM of DNA extracted from normal colon tissues, weak emission responses of up to 1.3-fold of the initial emission intensity were detected; in contrast, the emission intensity increased in a concentration-dependent manner in the presence of 0–1.6 μM of DNA extracted from colon tumour tissues, showing up to a 5.5-fold increase in emission at 1.9 μM. Thus, there was a difference between the emission responses of **2** to the DNA of human colon tumour tissue and the normal adjacent tissue.

In conclusion, we have identified two classes of luminescent Pt(II) complexes that can be used for the detection of DNA mismatches and abasic DNA sites. These Pt(II) complexes are able to differentiate between cells with different levels of MMR activity. Importantly, the differential emission responses of the Pt(II) complexes to DNA from human colon cancer tissues and normal colon tissue suggest that the Pt(II) complexes are promising candidates for tumour diagnosis via simple emission spectroscopy measurements of DNA extracts. The selective targeting of DNA mismatches also lends the complexes therapeutic potential for cancer treatment^21^; and they may be used as scaffolds in the design of new anti-cancer agents for the treatment of MMR-deficient cancers.

## Methods

### General

The synthesis and characterization of the platinum complexes and the experimental details of X-ray crystallographic analysis, emission titration experiments, ultraviolet/visible absorption titration experiments, ITC, ^1^H NMR titration experiments, melting temperature experiments and molecular docking model experiments are provided in the Supplementary Methods. The nucleic acid sequences for the different experiments are listed in the Supplementary Information.

### Quantum mechanics/molecular mechanics

The structures of mismatched DNA and complex **2** were taken from the literature (PDB ID: 2O1I^56^, unit Å) and the X-ray crystal analysis (CCDC 1061095), respectively. In general, molecular docking studies were performed using ICM-pro software to obtain the initial structure of DNA–complex **2** adducts^57^; subsequently, the binding between the DNA and complex was further optimized using the QM/MM approach and NWChem software^58^. The quantum part (QM) included complex **2** (total 133 atoms), and the rest of the system was modelled at the MM level. The QM region was treated at the density functional theory level using the M06L method^59^. The 6-31G* Pople basis set^60^ was employed for C, H, N and P atoms, and for the Pt atom, we applied the LANL2DZ^61^ basis set with effective core potential. The mismatched DNA was described with the AMBER parm99 force field^62^. Van der Waals parameters for the Pt atom were taken from the literature^63^. The docked structure was solvated in a 65-Å cubic box with 8,830 classical SPC/E water molecules. Twenty-two-positive (Na^+^) counterions were added to neutralize the charges in the system. The structure was optimized using the BFGS algorithm for the QM part and the steepest descent algorithm for the MM part. The optimization of these two regions (QM and MM) was alternated until self-consistency was reached.

### DNA extraction from cancer cells or tumour tissues

HCT116 and HCT116N cell lines were generously provided by Thomas A. Kunkel (NIH, USA). The human tissue samples were obtained from Jinan University, Guangzhou, China, and the experimental uses were authorized by the Human Experimentation Ethics Committee of Jinan University. Genomic DNA from cells or tissues was isolated using DNAzol Reagent (Thermo Fisher ) according to the manufacturer's protocol. For DNA extraction from cell lines, 1 ml of DNAzol Reagent was added to 1–3 × 10^7^ cells grown on 10-cm^2^ culture plates. Then, the cells were lysed by agitating the culture plate and gently pipetting the lysate into a microfuge tube. For the tissue sample, 1 ml of DNAzol Reagent was added to ∼50 mg of tissues. Then, the tissues were homogenized by applying three strokes in the homogenizer. The homogenate was sedimented for 10 min at 10,000*g* at 4 °C. Following centrifugation, the resulting viscous supernatant was transferred to a fresh tube, mixed with 0.5 ml of 100% ethanol by inversion and stored at room temperature for 1–3 min. Then, the DNA precipitate was washed twice with 1.0 ml of 75% ethanol. The DNA was air-dried and dissolved in 200 μl of 8 mM NaOH, and the pH was adjusted to 7.5 by the addition of HEPES. The emission spectrum of a solution of complex **1c** or **2** (5 μM) in buffer solution (50 mM NaCl and 2 mM Tris, pH 7.5) was recorded and titrated with increasing amounts of the isolated DNA. The emission spectra were recorded after equilibration for 3 min in each addition.

### Fluorescence microscopy examination

Cells (HCT116 or NCM460) were seeded in glass bottom culture dishes (35 mm) at a density of 1.0 × 10^5^ ml^−1^. After 12 h, the dishes were placed on ice, washed twice with 1 ml of cold PBS and then incubated in 1 ml of permeabilization buffer (20 mM HEPES, pH 7.5, 110 mM potassium acetate, 5 mM magnesium sulphate and 250 mM sucrose) containing 40 μg ml^−1^ digitonin for 30 min. After removal of the digitonin solution, the dishes were washed three times with cold permeabilization buffer for 1, 5 and 10 min. The cells were then incubated with complex 2 (10 μΜ) in transport buffer (20 mM HEPES, pH 7.3, 110 mM potassium acetate, 5 mM sodium acetate, 2 mM magnesium sulphate and 250 mM sucrose) for 15 min. Cells were directly subjected to fluorescence imaging without removing the transport buffer. For co-culture experiments, HCT116 cells were first seeded in culture dishes for 12 h. Next, the dishes were treated with a non-toxic concentration of Hoechst 33342 (1 μg ml^−1^) for 30 min and washed twice with PBS. Then, NCM460 cells were seeded in the same dishes for another 12 h. Finally, the co-cultured cells were incubated in permeabilization buffer, stained with complex **2** and subjected to fluorescence imaging as aforementioned.

## Additional information

**How to cite this article:** Fung, S. K. *et al*. Luminescent platinum(II) complexes with functionalized N-heterocyclic carbene or diphosphine selectively probe mismatched and abasic DNA. *Nat. Commun.* 7:10655 doi: 10.1038/ncomms10655 (2016).

## Supplementary Material

Supplementary Information Supplementary Figures 1-30, Supplementary Tables 1-2, Supplementary Methods and Supplementary References

## Figures and Tables

**Table 1:** A comparison of structure–emission response relationships of [Pt(ĈN̂N)(NHC)]CF_3_SO_3_ complexes containing *N*-benzyl groups towards matched and mismatched DNA.

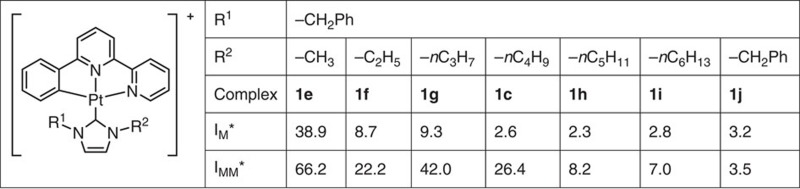								

*I_M_ and *I*_MM_ denote the relative emission intensities of the Pt(II) complexes towards matched and mismatched DNA, respectively.
