# Supplementary material for: Luminescent platinum(II) complexes with functionalized N-heterocyclic carbene or diphosphine selectively probe mismatched and abasic DNA
Source: Nat Commun. 2016 Feb 17;7:10655. doi: 10.1038/ncomms10655 (PMC4757794; doi:10.1038/ncomms10655)
Supplement: Supplementary Information — Supplementary Figures 1-30, Supplementary Tables 1-2, Supplementary Methods and Supplementary References [file ncomms10655-s1.pdf]

Supplementary Figures/Schemes/Tables

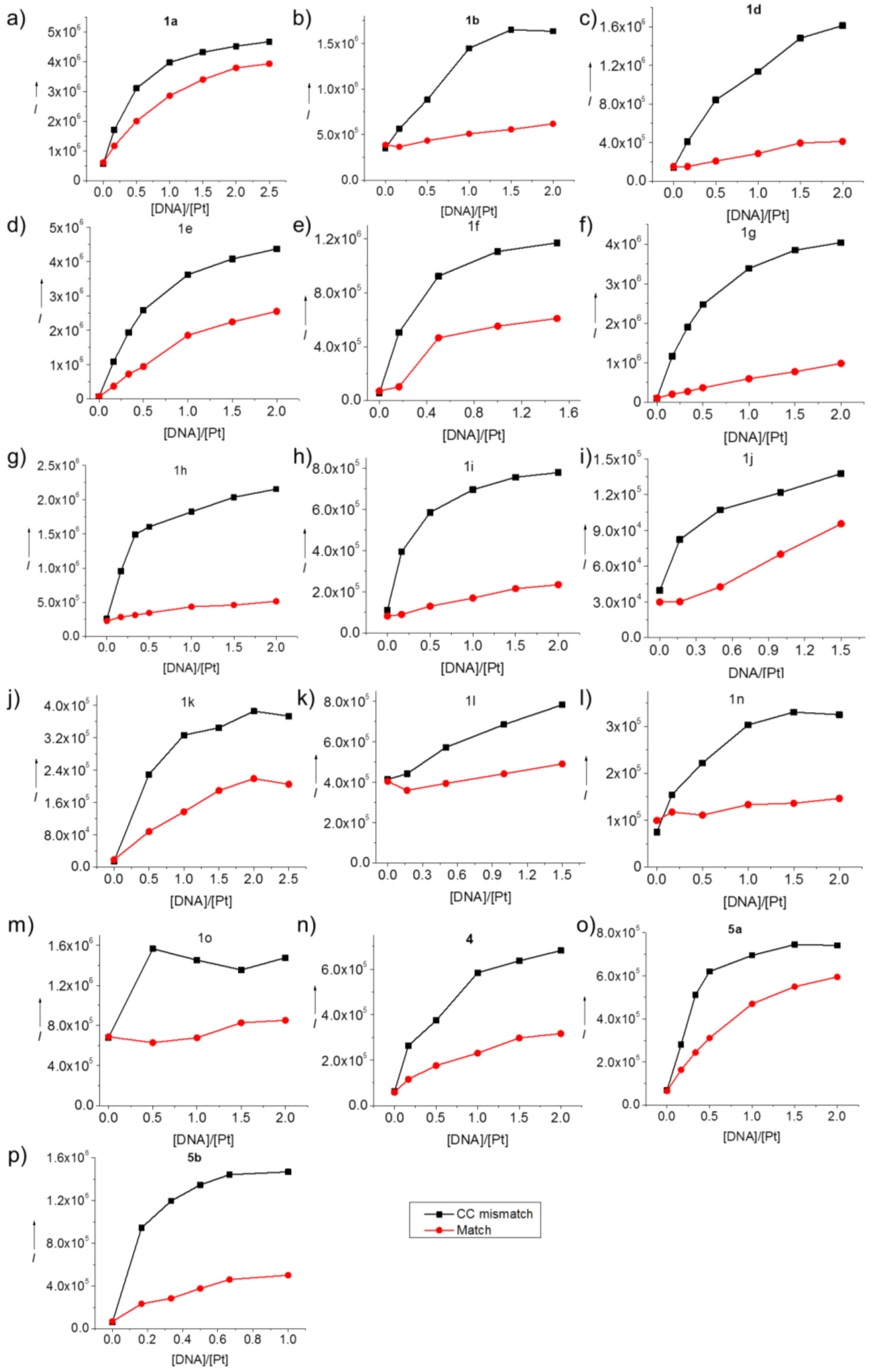

Supplementary Figure 1. Emission intensity of different Pt(II) complexes towards hairpin CC mismatched DNA and matched DNA.

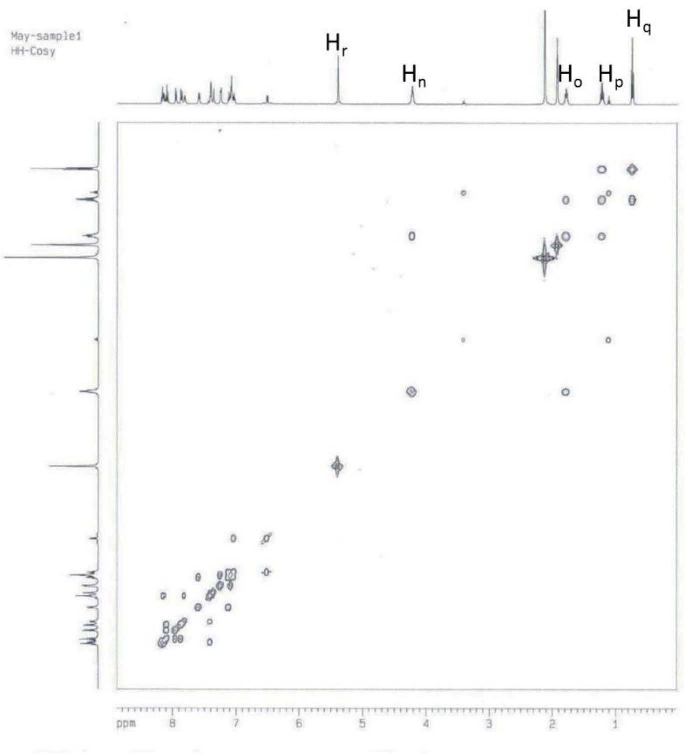
May-sample1
HH-Cosy
Hr
Hn
Ho
Hp
Hq
ppm

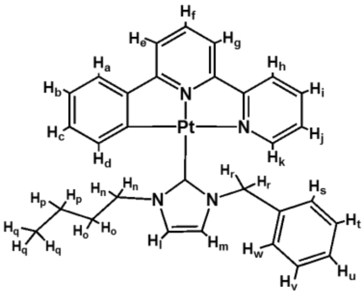
Pt
N
N
N
N
Ha
Hb
Hc
Hd
He
Hf
Hg
Hh
Hi
Hj
Hk
Hl
Hm
Hn
Ho
Hp
Hq
Hr
Hs
Ht
Hu
Hv
Hw

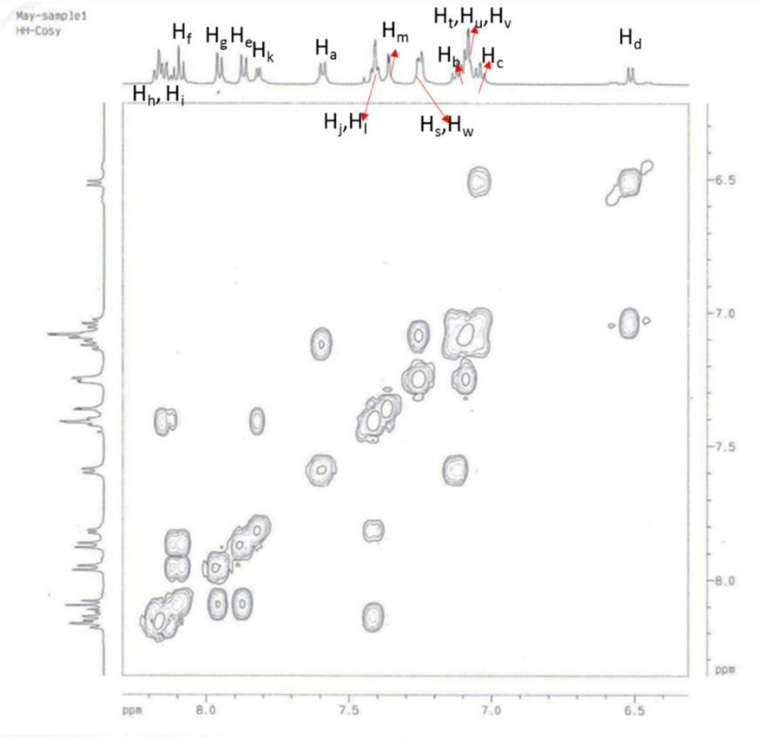
May-sample1
HH-Cosy
Hf
Hg
He
Hk
Ha
Hm
Ht,Hu,Hv
Hb
Hc
Hd
Hh, Hi
Hj,Hl
Hs,Hw
ppm

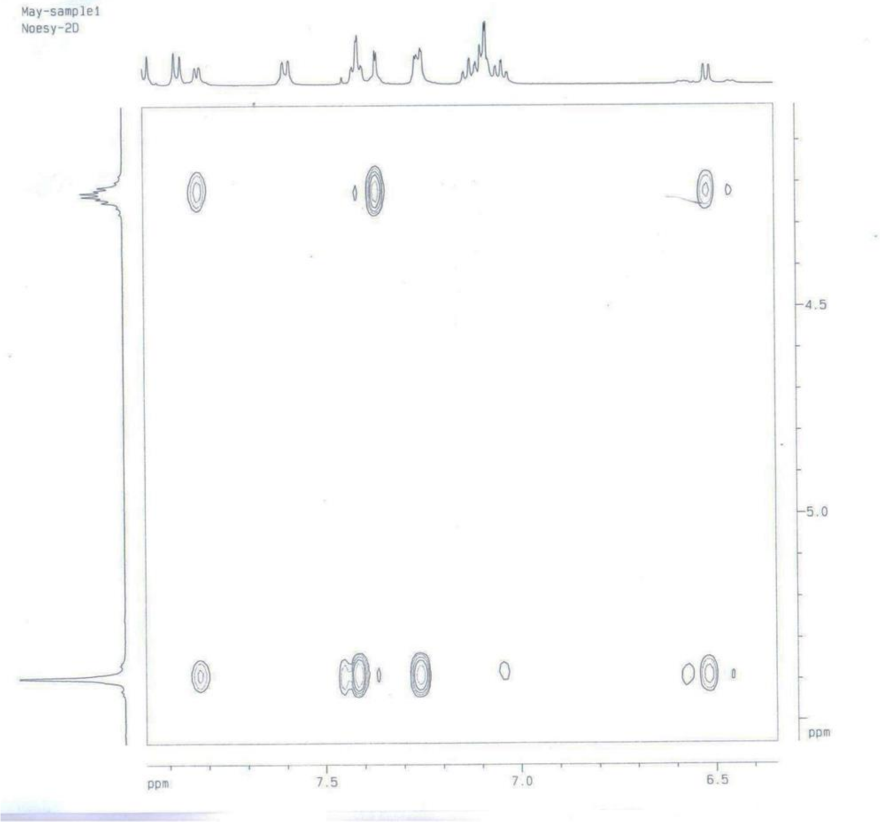

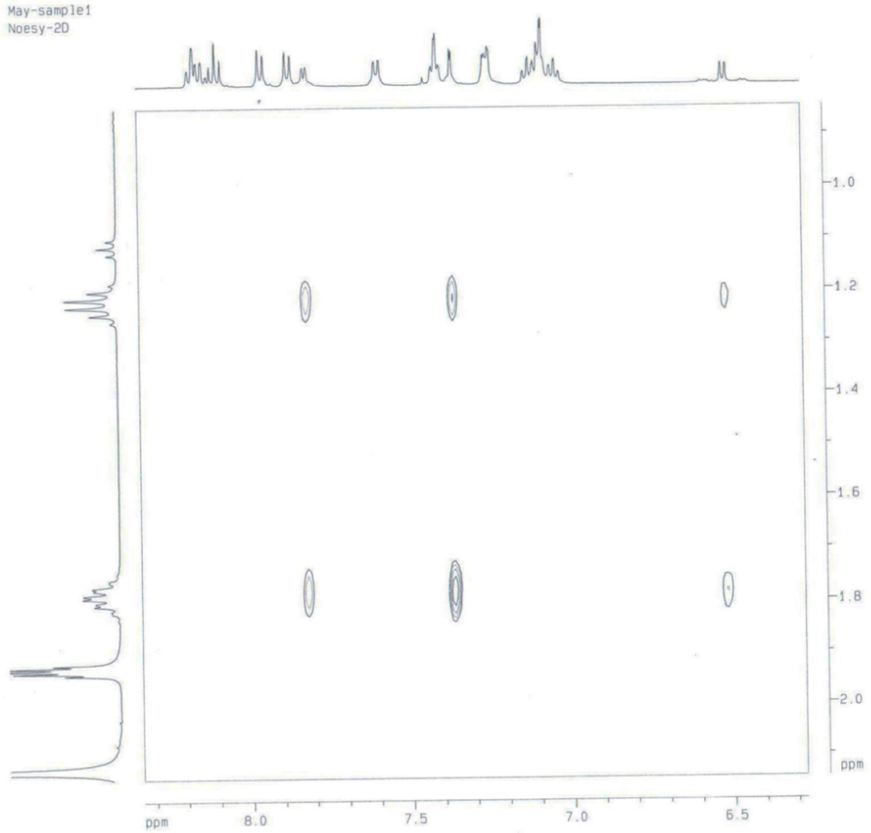

Supplementary Figure 2. ^{1}H–^{1}H COSY and NOESY NMR spectrum of **1c**.

a)

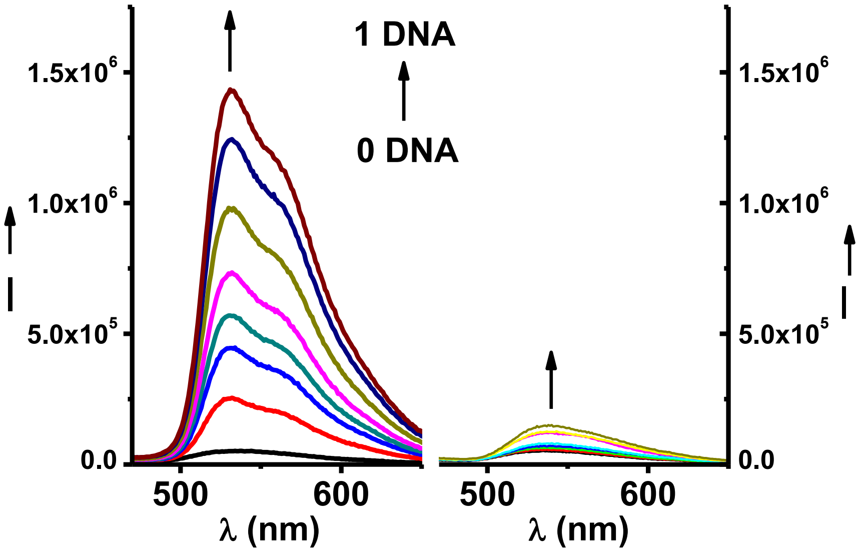

b)

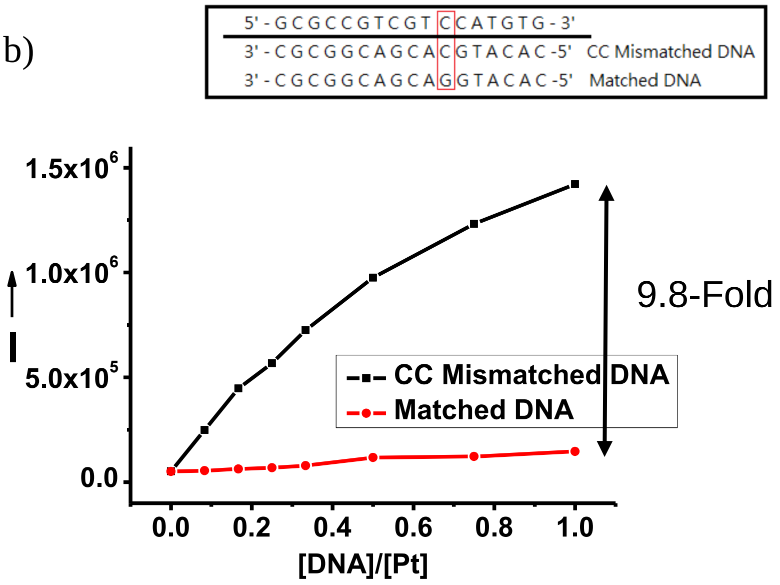

Supplementary Figure 3. (**a**) The emission spectra of complex **1c** (5 µM) in an aqueous buffer solution (50 mM NaCl, 2 mM Tris, pH 7.5) after binding to different concentrations of CC mismatched DNA (left) and well-matched DNA (right). (**b**) Changes in emission intensity at 535 nm of complex **1c** (5 µM) in the aqueous buffer solution upon addition of different types of DNAs. Inset shows the sequences of double strand DNA.

(a)

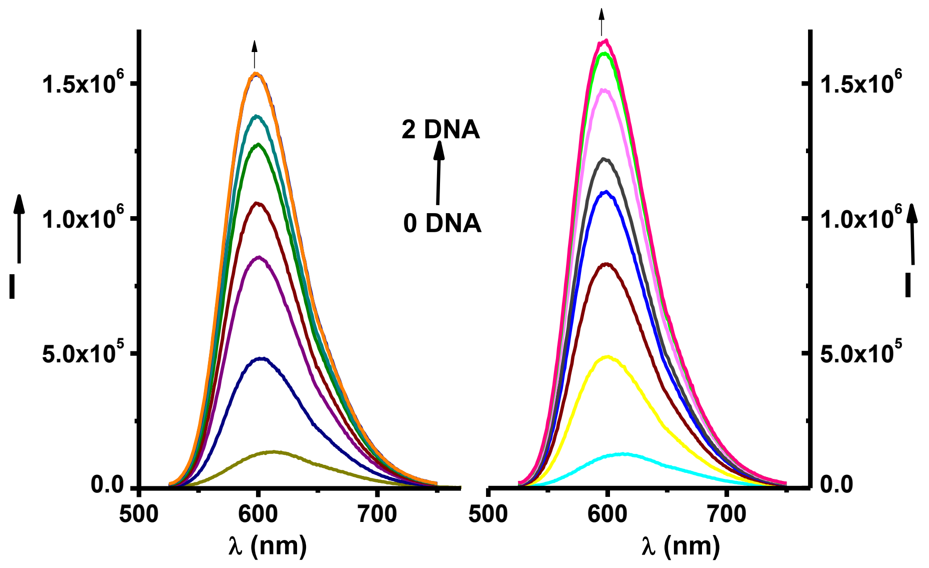

(b)

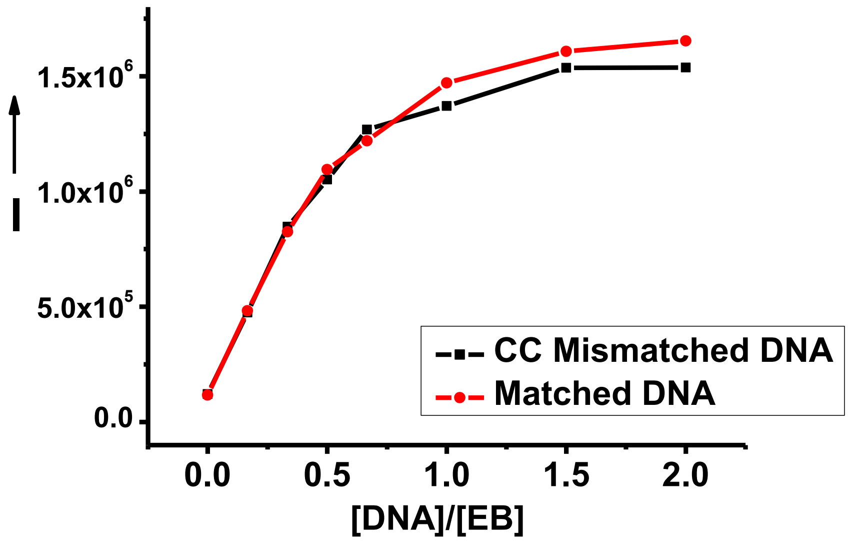

Supplementary Figure 4. (**a**) The emission spectra of Ethidium Bromide (EB) (5 μM) in an aqueous buffer solution (50 mM NaCl, 2 mM Tris, pH 7.5) after binding to different concentrations of CC mismatched DNA (left) and well-matched DNA (right). (**b**) Changes in emission intensity at 597 nm of EB (5 μM) in the aqueous buffer solution upon addition of different types of DNAs.

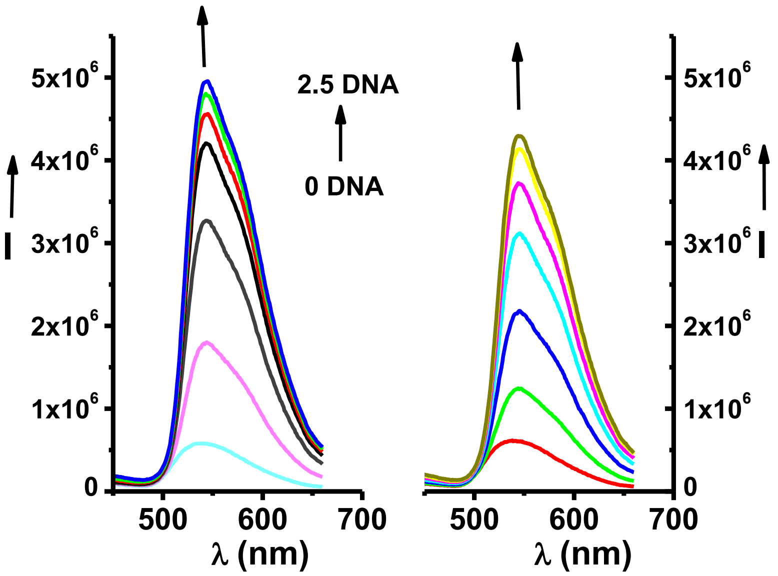

Supplementary Figure 5. The emission spectra of complex **1a** (5 μM) in an aqueous buffer solution (50 mM NaCl, 2 mM Tris, pH 7.5) after binding to different concentrations of CC mismatched DNA (left) and well-matched DNA (right).

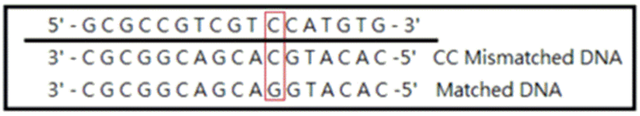

(a)

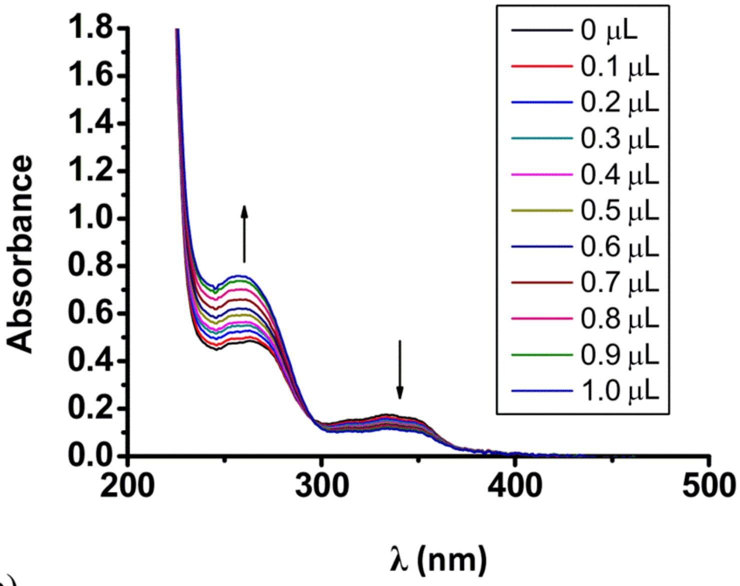

(b)

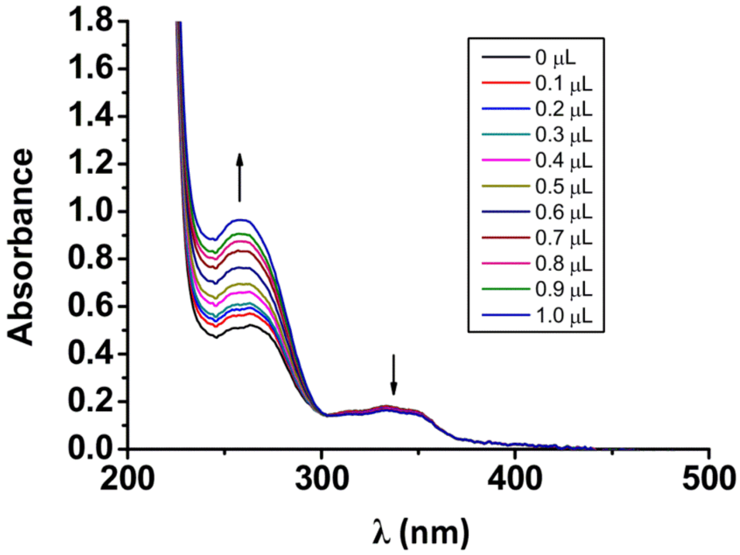

Supplementary Figure 6. (**a**) UV-vis spectra of **1c** (20 μM) in an aqueous buffer solution (50 mM NaCl, 2 mM Tris, pH 7.5) with increasing concentration of CC mismatched DNA at 298 K. Inset shows the sequences of double strand DNA. (**b**) UV-vis spectra of **1c** (20 μM) in an aqueous buffer solution (50 mM NaCl, 2 mM Tris, pH 7.5) with increasing concentration of matched DNA at 298 K.

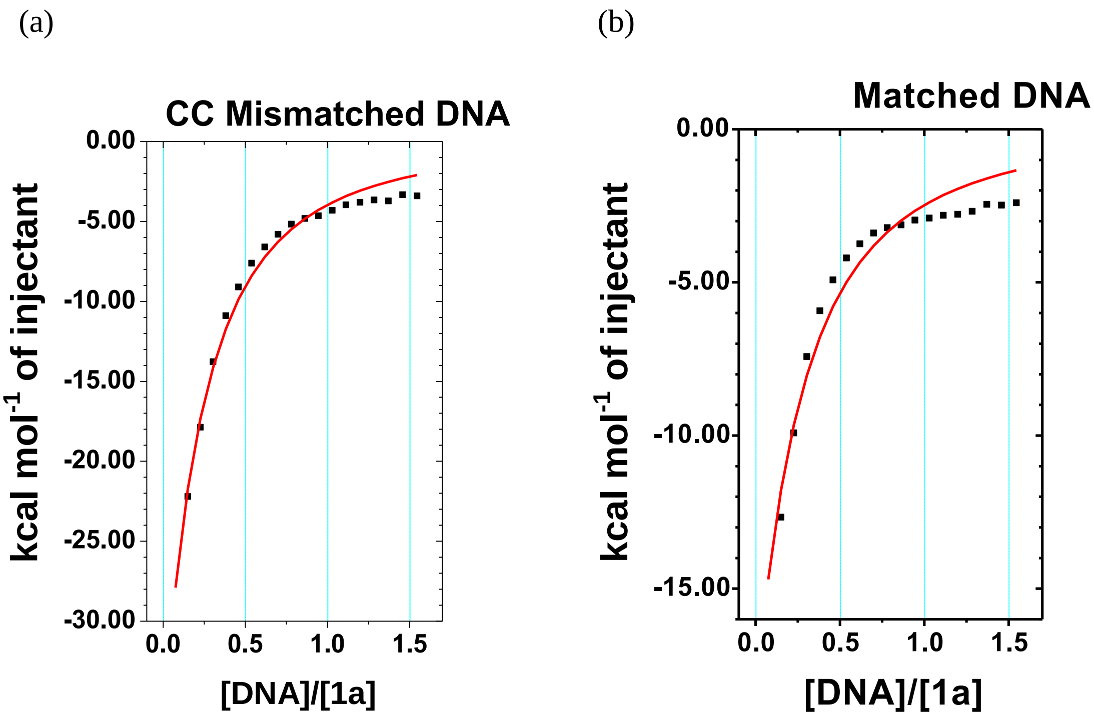

Supplementary Figure 7. (**a**) Plot of integrated ITC data for the exothermic interaction between CC mismatched DNA (0.75 mM) and **1a** (0.1 mM). The solid line represents the best least-squares fit to the data. (**b**) Plot of integrated ITC data for the exothermic interaction between matched DNA (0.75 mM) and **1a** (0.1 mM). The solid line represents the best least-squares fit to the data

(a) (b)

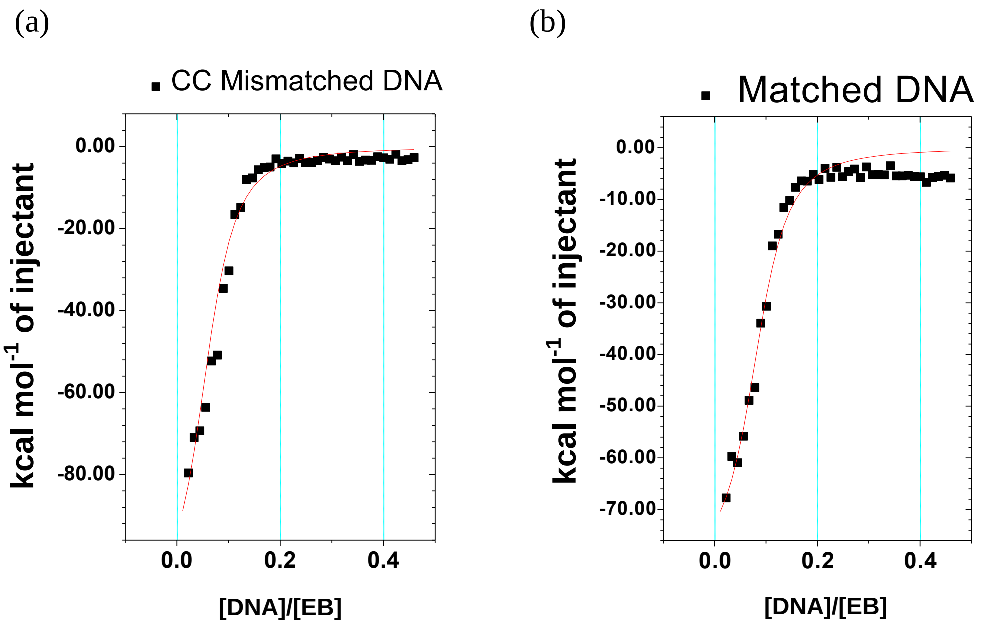

Supplementary Figure 8. (**a**) Plot of integrated ITC data for the exothermic interaction between CC mismatched DNA (0.75 mM) and **EB** (0.1 mM). The solid line represents the best least-squares fit to the data. (**b**) Plot of integrated ITC data for the exothermic interaction between matched DNA (0.75 mM) and **EB** (0.1 mM). The solid line represents the best least-squares fit to the data.

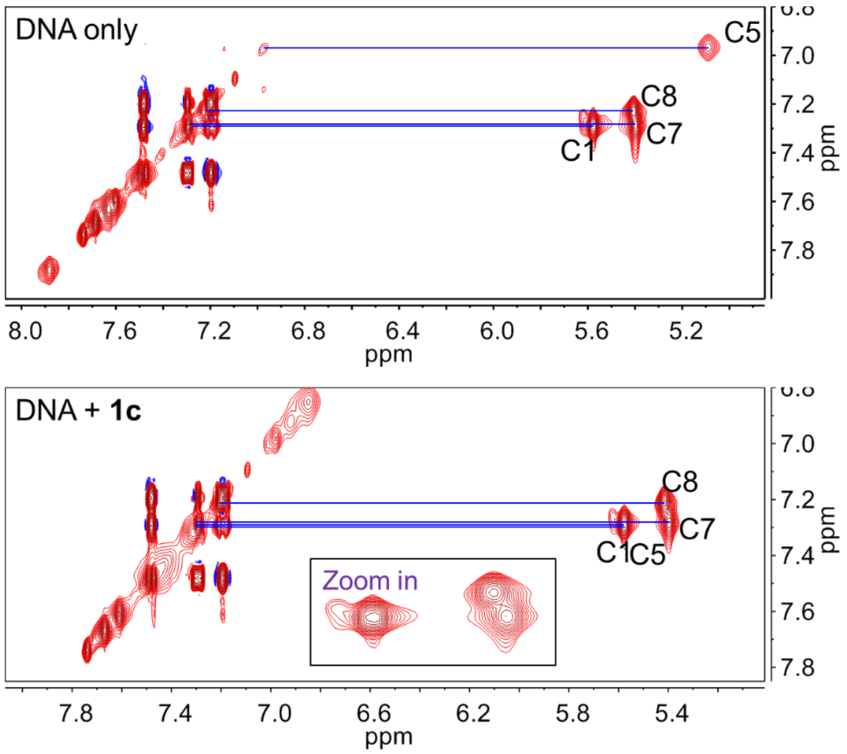

Supplementary Figure 9. ^{1}H-^{1}H TOCSY NMR (phosphate buffer = 50 mM, NaCl = 20 mM, in D_2O 10 ℃) spectra of self-complementary DNA oligonucleitide (5′-$C_1G_2G_3A_4\underline{C}_5T_6C_7C_8G_9$-3′) having a CC mismatch (top) and the DNA with **1c** in a 1:1 molar ratio (down).

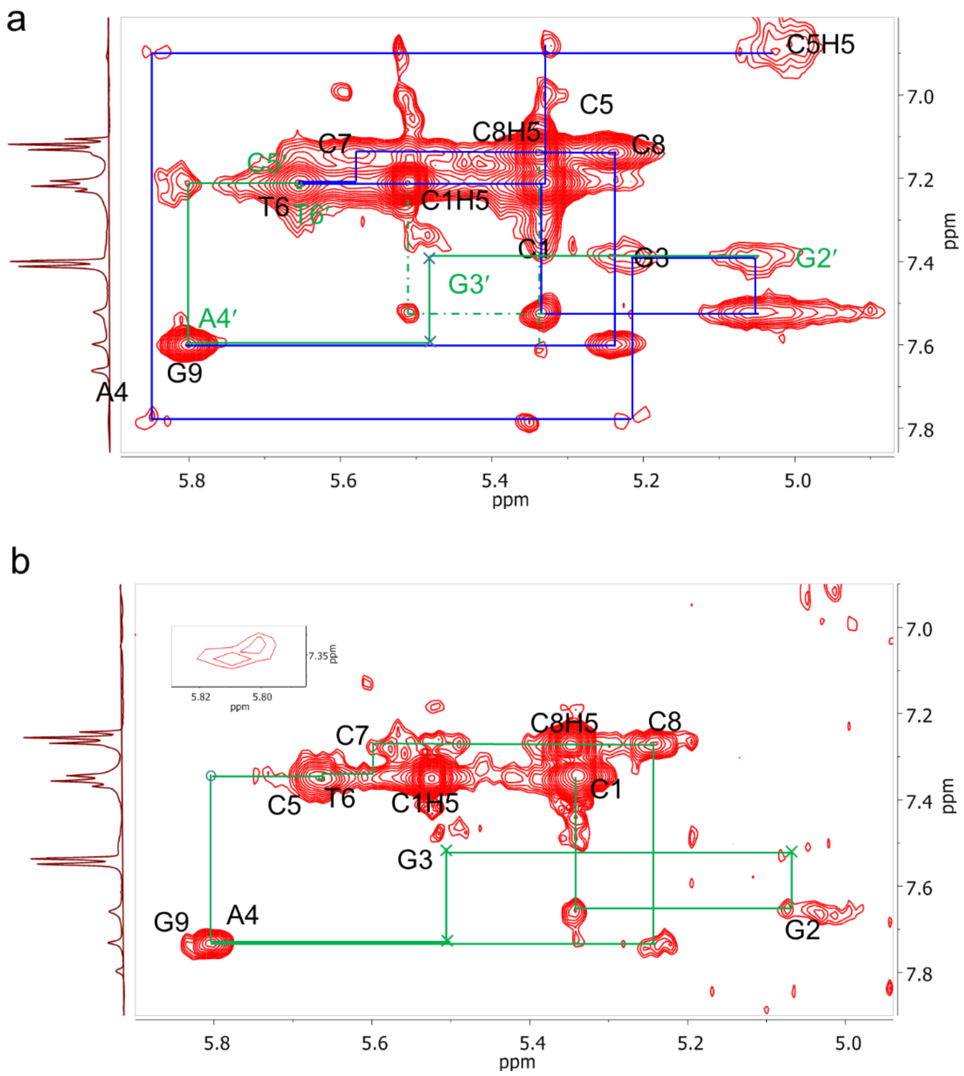

Supplementary Figure 10. ^{1}H-^{1}H NOESY NMR (phosphate buffer = 50 mM, NaCl = 20 mM, in D_2O 10 ℃) of the mixture of CC mismatched DNA with **1c** at ratio of 1:0.25 (**a**) and 1:0.5 (**b**).

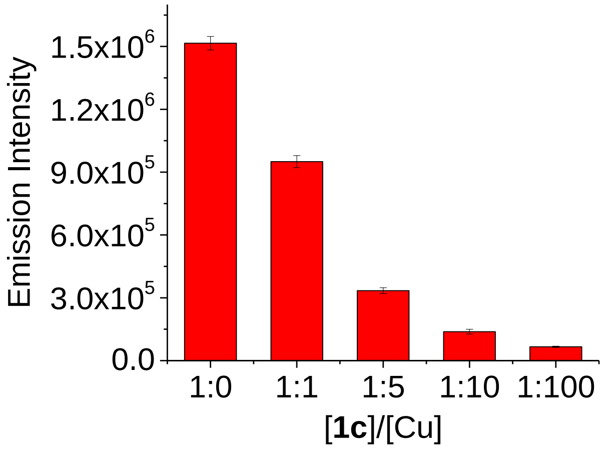

Supplementary Figure 11. The peak emission intensity of **1c** with equimolar of CC mismatched DNA in response to increasing amount of $[Cu(phen)_2]^{2+}$.

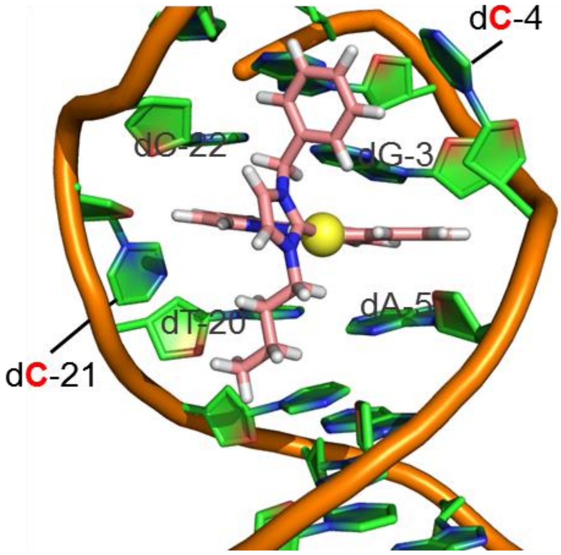

Supplementary Figure 12. Molecular docking models of **1c** bound with DNA containing CC mismatch.

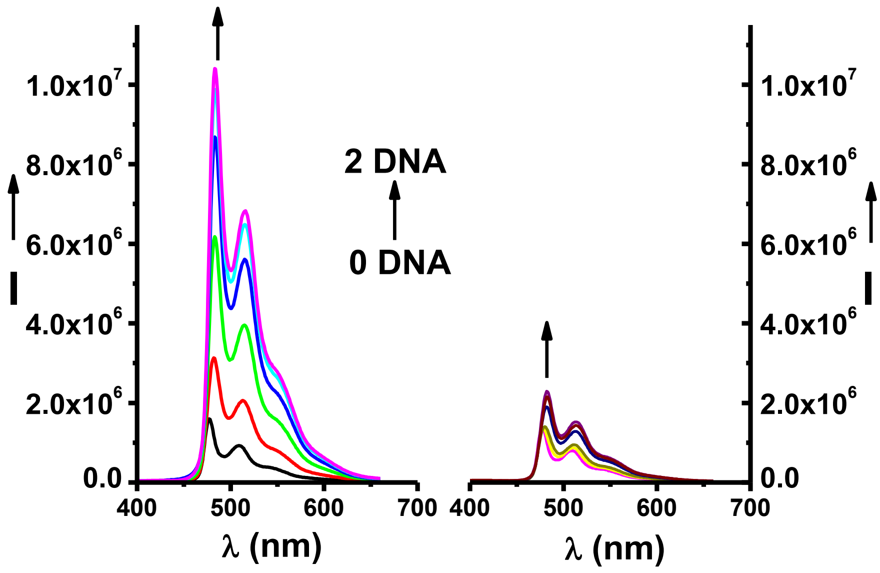

Supplementary Figure 13. The emission spectra of complex **1d** (5 μM) in an aqueous buffer solution (50 mM NaCl, 2 mM Tris, pH 7.5) after binding to different concentrations of CC mismatched DNA (left) and well-matched DNA (right).

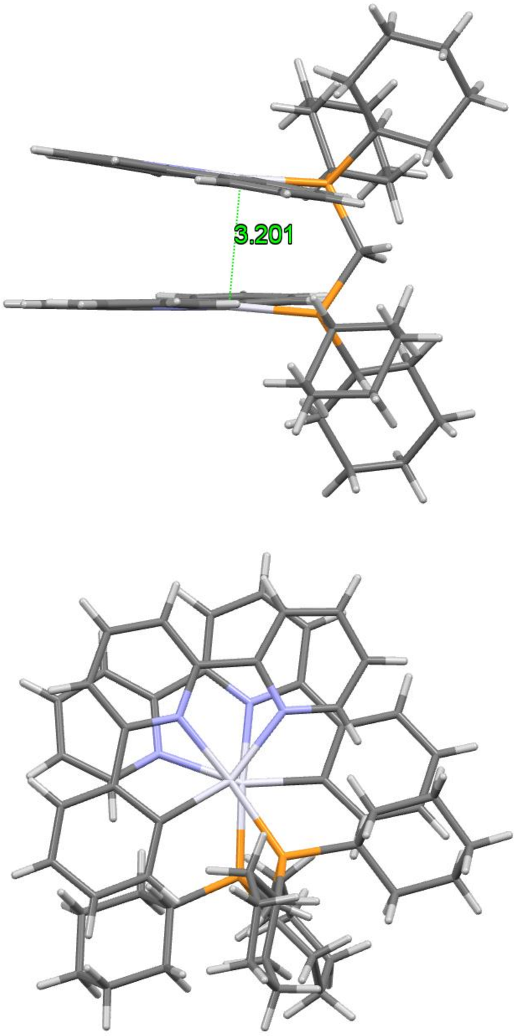

Supplementary Figure 14. The crystal structure of complex **2** showing intramolecular Pt-Pt interactions.

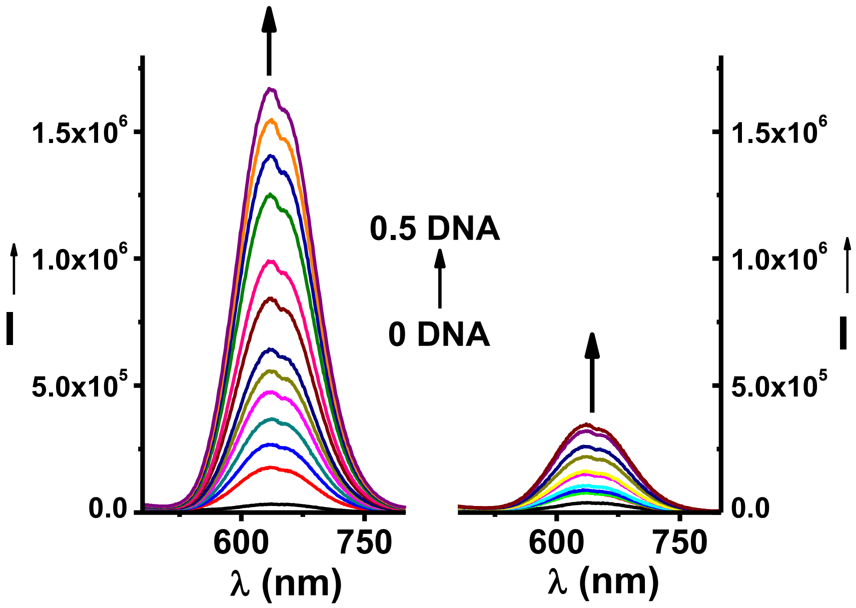

Supplementary Figure 15. The emission spectra of complex **2** (5 μM) in an aqueous buffer solution (50 mM NaCl, 2 mM Tris, pH 7.5) after binding to different concentrations of CC mismatched DNA (left) and well-matched DNA (right).

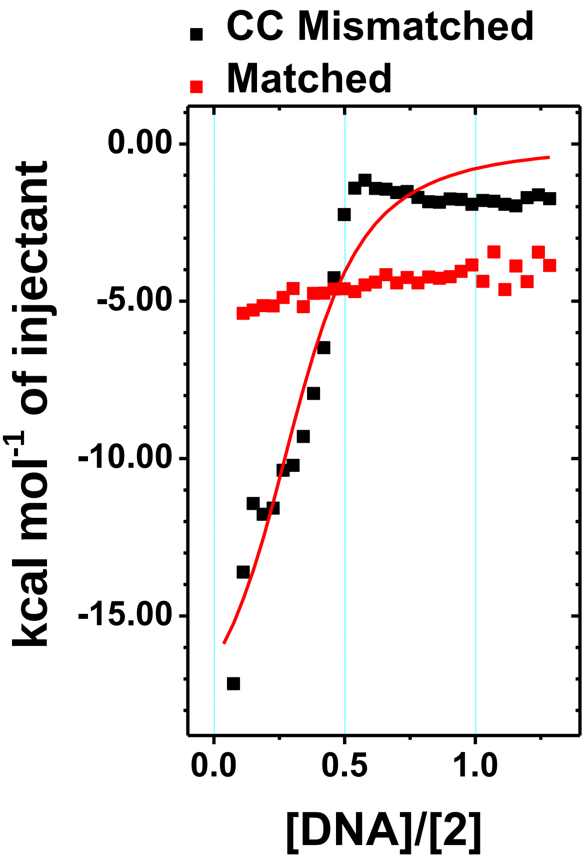

Supplementary Figure 16. Plot of integrated ITC data for the exothermic interaction between CC mismatched or matched DNA (0.75 mM) and complex **2** (0.1 mM). The solid line represents the best least-squares fit to the data.

(a)

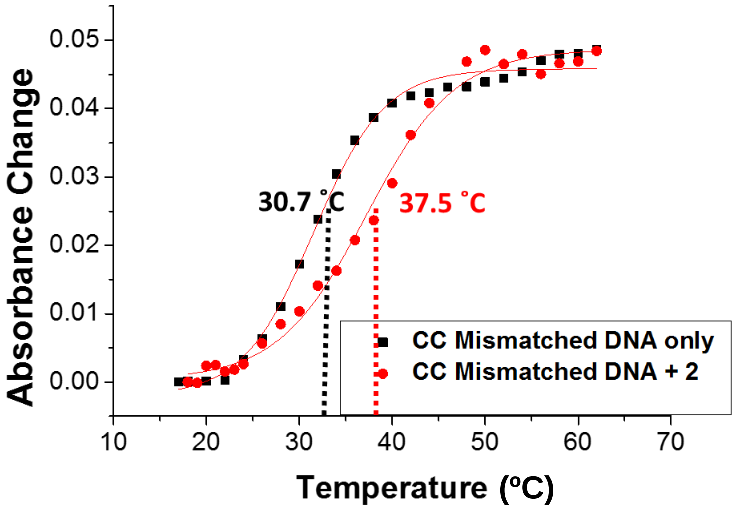

(b)

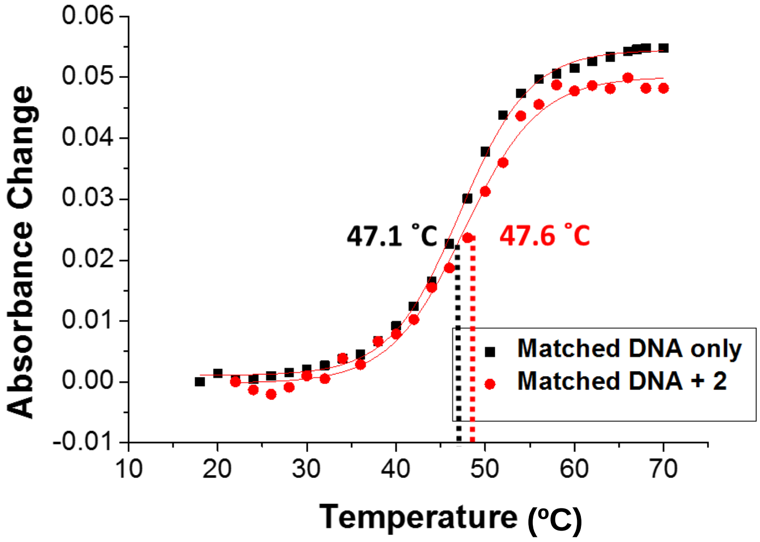

Supplementary Figure 17. (**a**) The change in UV-vis absorption at 260 nm of CC mismatched DNA (2 µM) with or without **2** (2 µM) upon increasing temperature. (**b**) The change in UV-vis absorption at 260 nm of matched DNA (2 µM) with or without **2** (2 µM) upon increasing temperature.

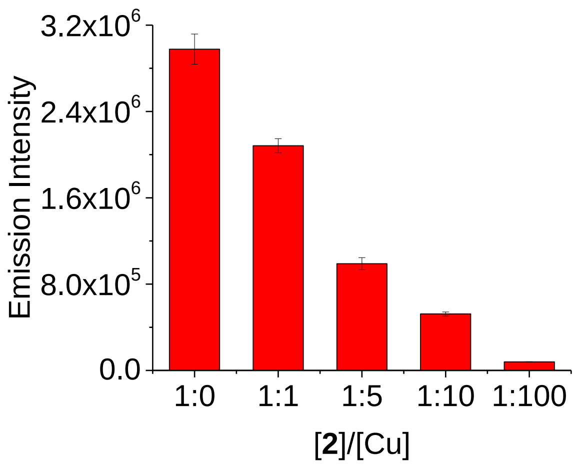

Supplementary Figure 18. The peak emission intensity of **2** with equimolar of CC mismatched DNA in response to increasing amount of $[Cu(phen)_2]^{2+}$.

(a)

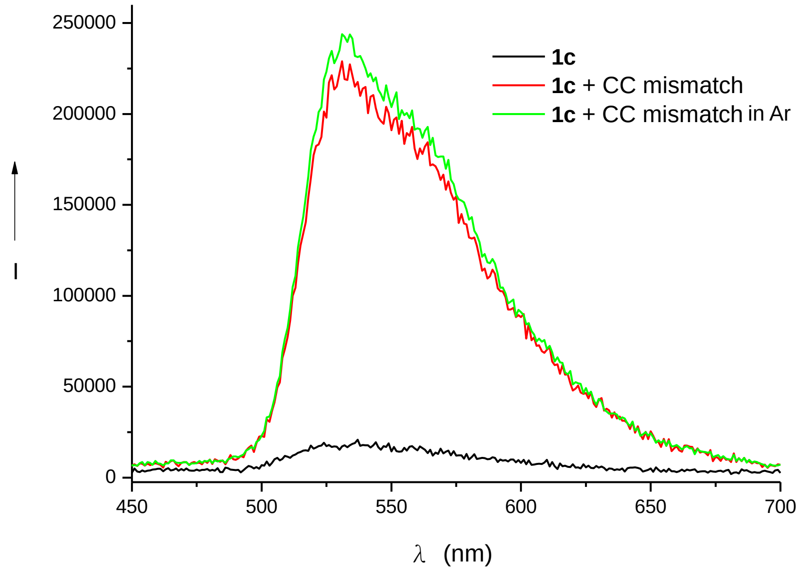

(b)

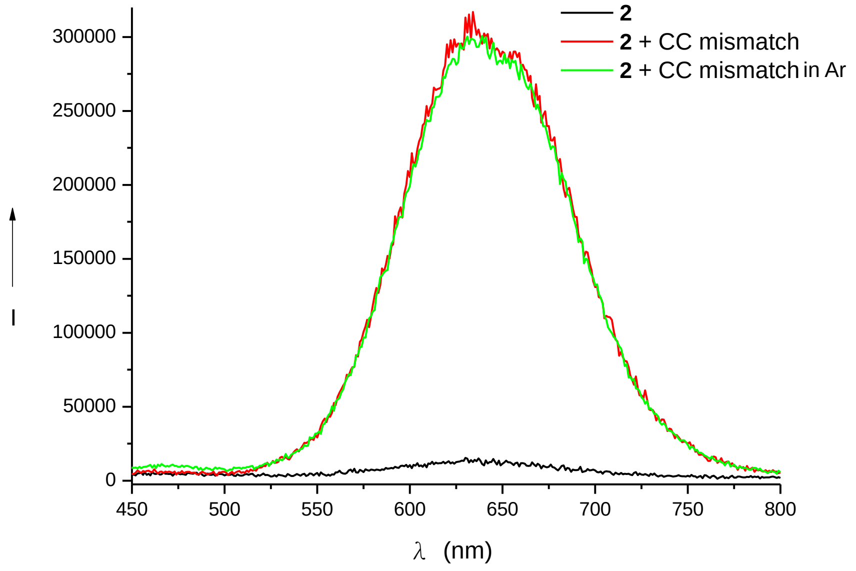

Supplementary Figure 19. Emission spectra of (**a**) **1c** or (**b**) **2** in the absence or in the presence of CC mismatched DNA in air or in Ar.

(a)

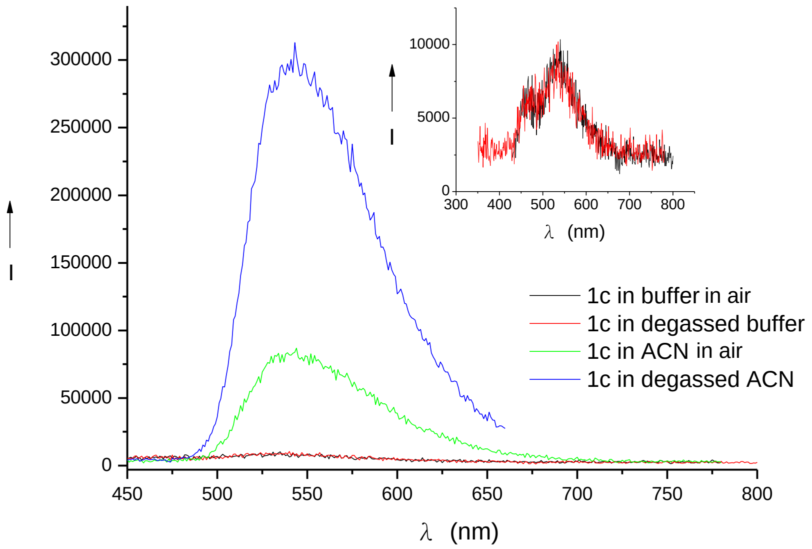

300000
250000
200000
150000
100000
50000
0
I
450
500
550
600
650
700
750
800
λ (nm)
10000
5000
0
I
300
400
500
600
700
800
λ (nm)
1c in buffer in air
1c in degassed buffer
1c in ACN in air
1c in degassed ACN

(b)

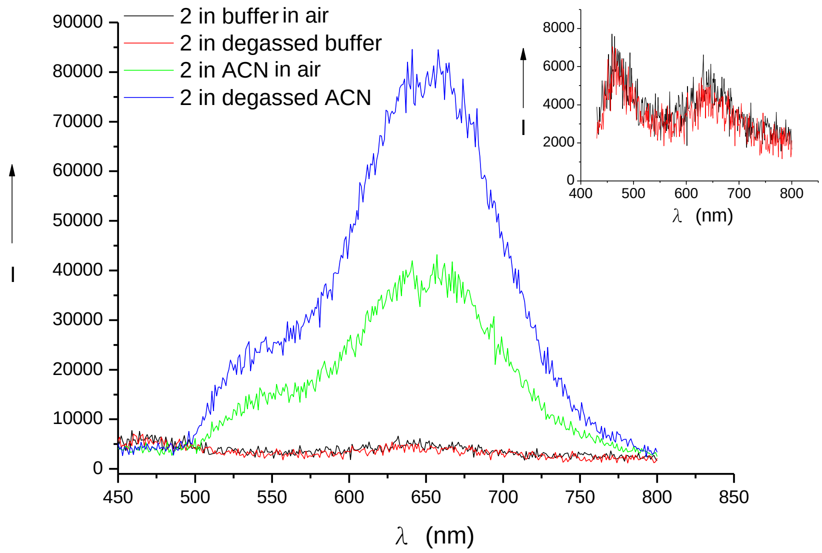

2 in buffer in air
2 in degassed buffer
2 in ACN in air
2 in degassed ACN
90000
80000
70000
60000
50000
40000
30000
20000
10000
0
I
450
500
550
600
650
700
750
800
850
λ (nm)
8000
6000
4000
2000
0
I
400
500
600
700
800
λ (nm)

(c)

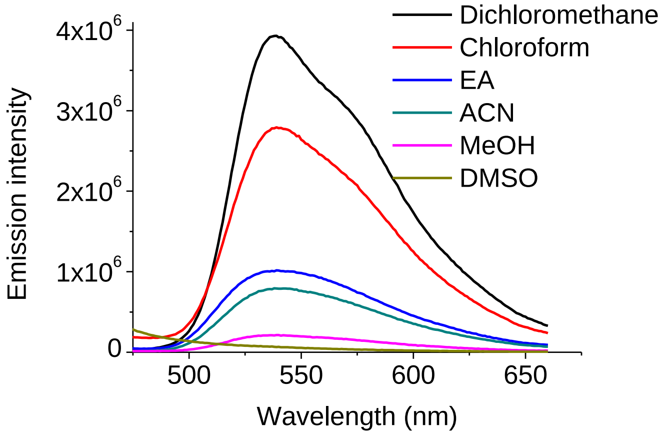

Supplementary Figure 20. (**a-b**) Emission spectra of **1c** (**a**) or **2** (**b**) in buffer or acetonitrile (ACN) solutions under air or degassed. (**c**) Emission spectra of **1c** in various organic solvents having different donor strength.

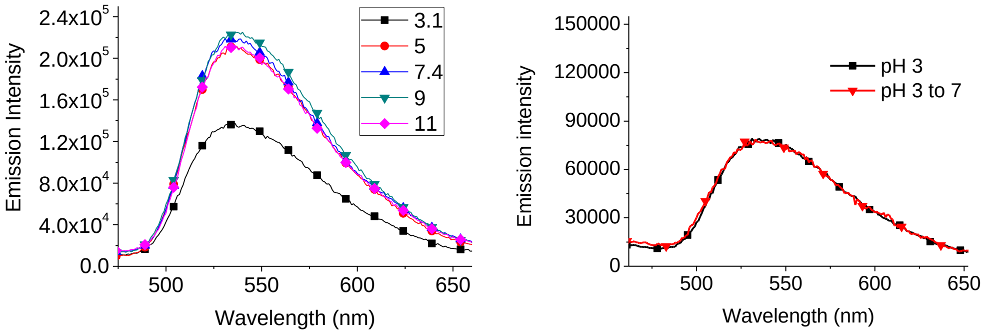

Supplementary Figure 21. Emission spectra of **1c** (10 μM) at different pH. From pH 5-11 (left), no significant change of emission spectra was found. At low pH 3.1, **1c** displayed lower emission intensity, which is caused by decomposition of the complex as the emission intensity cannot be recovered when adjusting the pH to 7 (right).

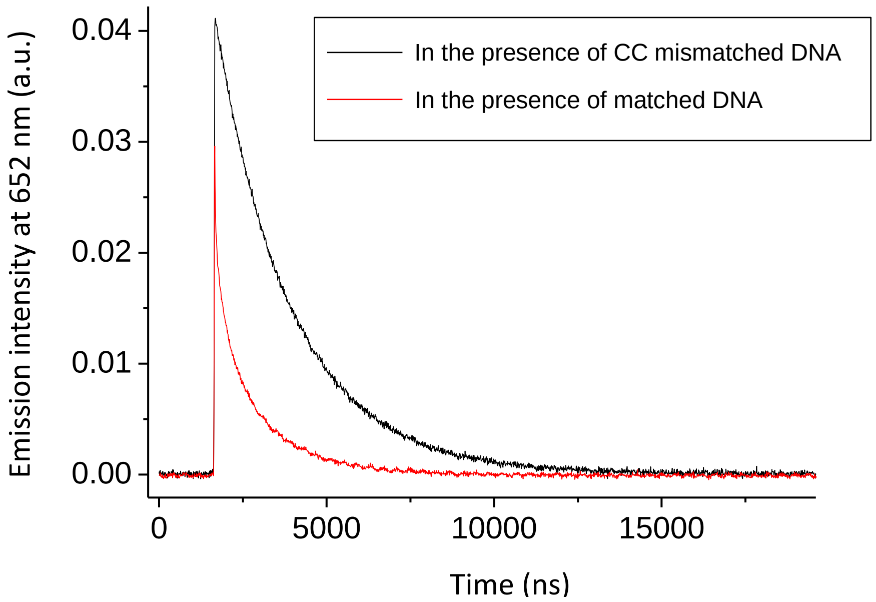

Supplementary Figure 22. Emission decay of **2** in the presence of CC mismatched DNA or matched DNA at 652 nm.

(a)

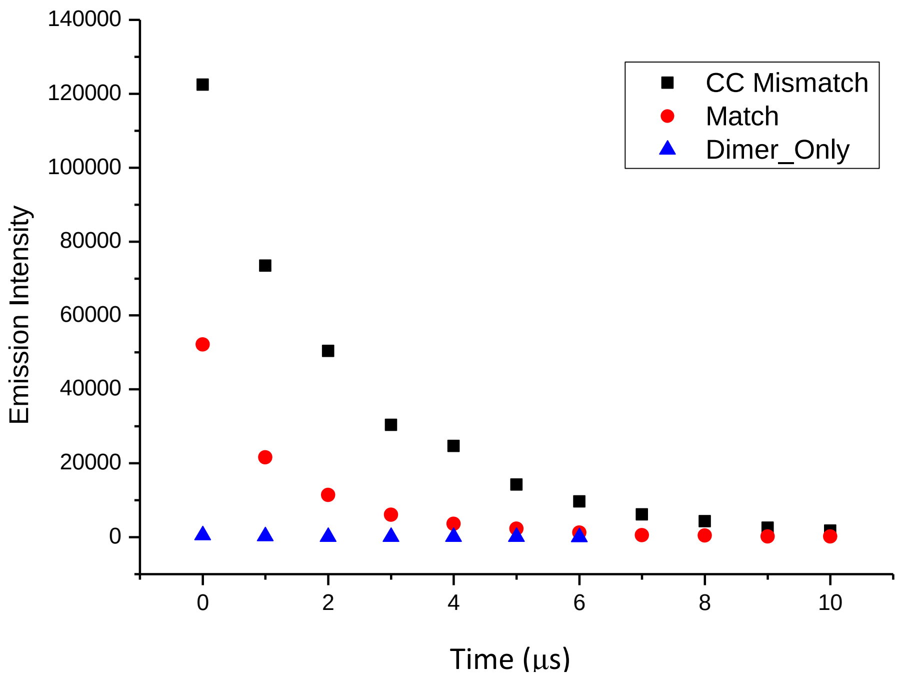

(b)

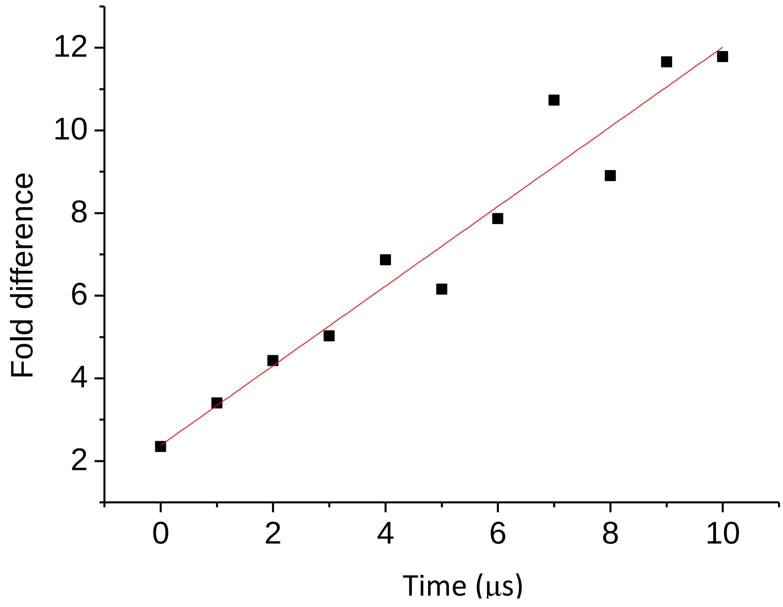

Supplementary Figure 23. (**a**) Peak (at 652 nm) emission intensity of **2** in the presence of CC mismatched DNA or matched DNA. (**b**) The ratio of $I_{mismatch}/I_{match}$ at different decay time.

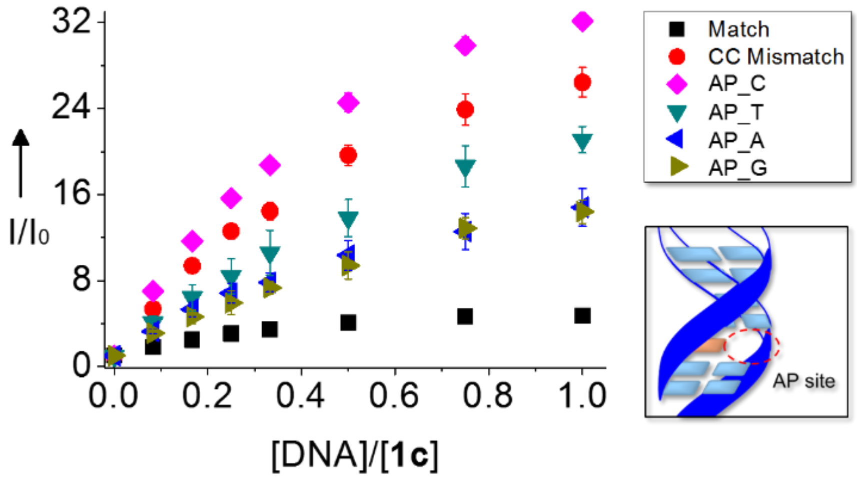

Supplementary Figure 24. The emission responses of **1c** (5 μM) at 535 nm in an aqueous buffer solution (50 mM NaCl, 2 mM Tris, pH 7.5) after addition of different concentrations of abasic DNA. The dsDNAs were obtained by annealing 3′-CTGGTCGAATAGTAXGGATCTATTCGC-5′ (X = C, CC mismatch; or X= abasic; or X = G, matched sequence) with its complementary DNA.

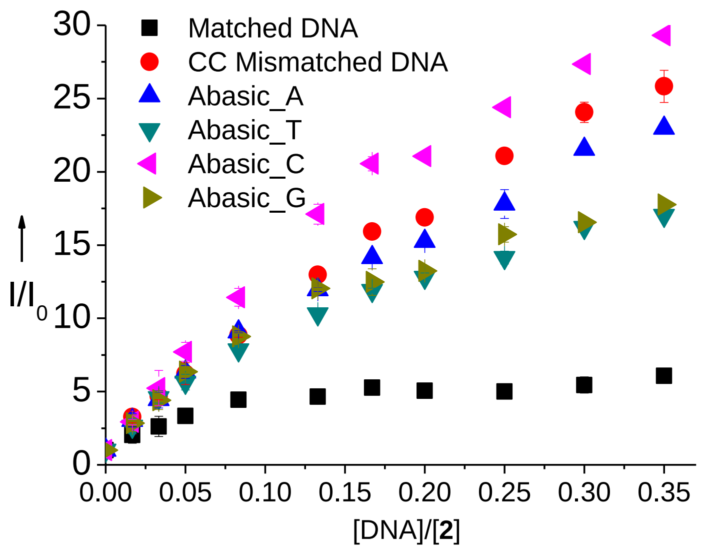

Supplementary Figure 25. The emission responses of **2** (5 μM) at 634 nm in an aqueous buffer solution (50 mM NaCl, 2 mM Tris, pH 7.5) after addition of different concentrations of abasic DNA.

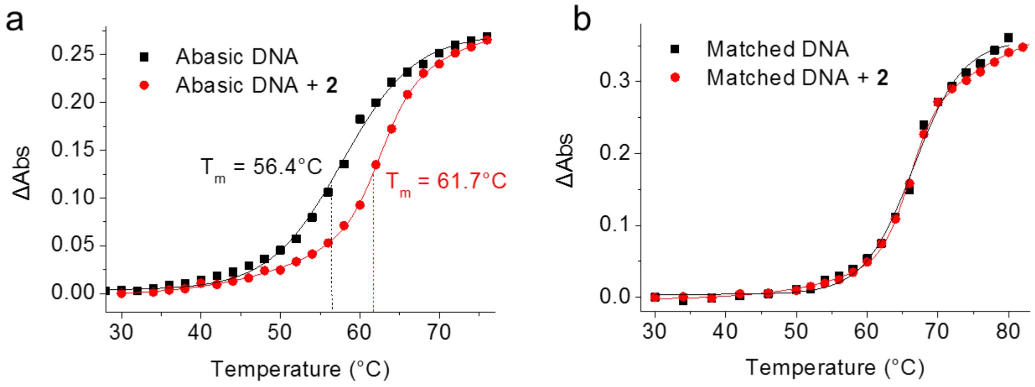

Supplementary Figure 26. Change of T_m (ΔAbs) of abasic DNA (**a**) and matached DNA (**b**) in the presence of complex **2**.

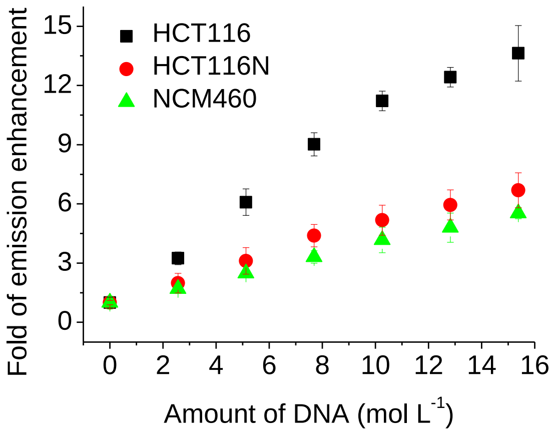

Supplementary Figure 27. Changes in emission intensity (± s.e.m.) of **2** (15 μM) at 634 nm in an aqueous buffer solution (50 mM NaCl, 2 mM Tris, pH 7.5) after incubation with different concentrations of DNA extracted from colon cell lines.

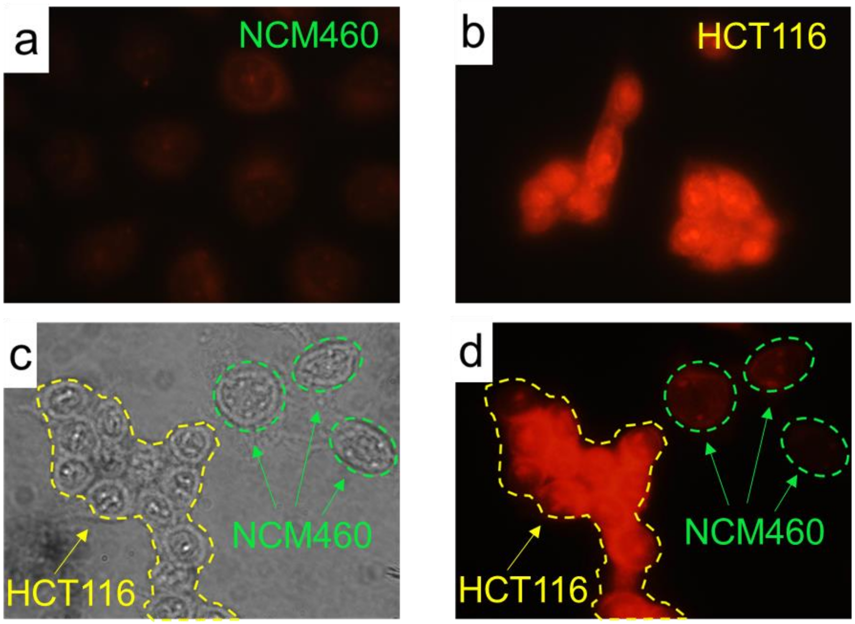

Supplementary Figure 28. Fluorescence microscopy of (**a**) NCM460 and (**b**) HCT116 cells pre-treated with digitonin and stained with 5 µM **2**. (**c**) Bright field and (**d**) fluorescence microscopy of HCT116 cells co-cultured with NCM460 and stained with **2**. HCT116 and NCM460 cells are indicated with yellow and green circle , respectively.

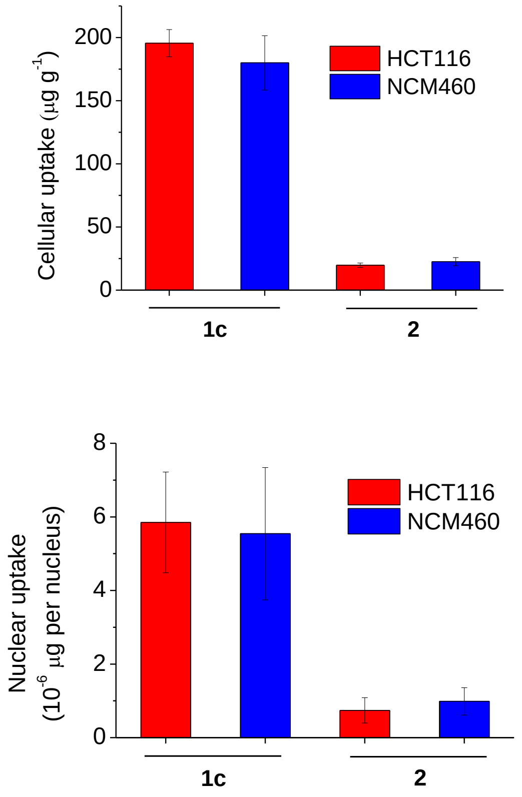

Supplementary Figure 29. ICP-MS experiments showing that both **1c** and **2** displayed similar cellular uptake of Pt in the whole cell (top) and in the nucleus (down) after 1 h treatment.

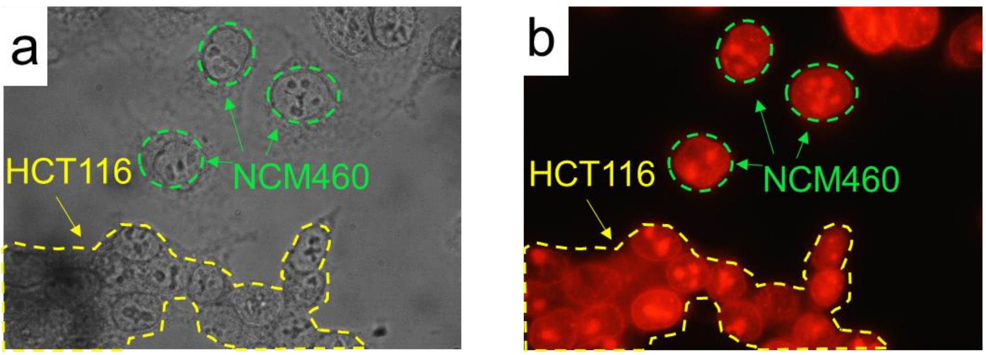

Supplementary Figure 30. Fluorescence microscopy of HCT116 cells co-cultured with NCM460 and stained with EB. Yellow and green circle indicates HCT116 and NCM460 cells, respectively.

Identification code	**2**
Empirical formula	$C_{58}H_{68}F_3N_4O_3P_2Pt_2S$*
Formula weight	1410.34
Temperature/K	100
Crystal system	monoclinic
Space group	P2/n
a/Å	12.8625(5)
b/Å	15.3569(6)
c/Å	16.0313(7)
α/°	90.00
β/°	102.4921(16)
γ/°	90.00
Volume/Å ^{3}	3091.7(2)
Z	2
ρ_{calc}g/cm^3	1.515
μ/mm^{-1}	9.567
F(000)	1394.0
Crystal size/mm^3	0.4 × 0.2 × 0.2
Radiation	CuKα (λ = 1.54178)
2Θ range for data collection/°	8.02 to 108.8
Index ranges	$-13 \leq h \leq 13$, $-16 \leq k \leq 16$, $-16 \leq l \leq 16$
Reflections collected	17745
Independent reflections	3799 [R_{int} = 0.0471, R_{sigma} = 0.0366]
Data/restraints/parameters	3799/291/330
Goodness-of-fit on F^2	1.076
Final R indexes [I>=2σ (I)]	R_1 = 0.0320, wR_2 = 0.0855
Final R indexes [all data]	R_1 = 0.0364, wR_2 = 0.0876
Largest diff. peak/hole / e Å $^{-3}$	1.08/-0.92

* Satisfactory disorder models for the solvent and another triflic counteranion of **2** were not found, and therefore the OLEX2 Solvent Mask routine (similar to PLATON/SQUEEZE) was used to mask out the disordered density.

Supplementary Table 1. Crystal data and structure refinement for complex **2**.

Experiment	DNA Sequence
Emission titration experiment (Hairpin)	T-T-C-G-G-T-A-X-G-G-A-C-G-G—5'<br>T<br>T-T-G-C-C-A-T-Y-C-C-T-G-C-C—3'<br><br>X Y<br>A T<br>C G (Matched DNA)<br>C C<br>A C<br>T C<br>A A<br>T T<br>G A<br>G T<br>G G (Mismatched DNA)
Emission titration experiment (double strand)	5'- GCG CCG TCG TCC ATG TG -3'<br>3'- CGC GGC AGC AGG TAC AC -5' Matched DNA<br>3'- CGC GGC AGC ACG TAC AC -5' CC Mismatched DNA
UV-visible absorption titration experiment	5'- GCG CCG TCG TCC ATG TG -3'<br>3'- CGC GGC AGC AGG TAC AC -5' Matched DNA<br>3'- CGC GGC AGC ACG TAC AC -5' CC Mismatched DNA
Emission titration experiment to CC mismatched DNA with different adjacent base pairs	5'—G-G-C-A-G-X-C-Y-T-G-G-C-T-T-T<br>3'—C-C-G-T-C-X'-C-Y'-A-C-C-G-T-T<br><br>X, X' and Y, Y' are matched base pairs
Isothermal titration calorimetry (ITC) experiment	5'- GCG CCG TCG TCC ATG TG -3'<br>3'- CGC GGC AGC AGG TAC AC -5' Matched DNA<br>3'- CGC GGC AGC ACG TAC AC -5' CC Mismatched DNA
NMR titration experiment	5'- CGG ACT CCG -3'<br>3'- GCC TCA GGC -5'
Melting temperature experiment	5'- ATC GTC TCG GTA TAA -3'<br>3'- TAG CAG AGC CAT ATT -5' Matched DNA<br>3'- TAG CAG ACC CAT ATT -5 CC Mismatched DNA
Emission titration experiment (abasic site)	5'- GAC CAG CTT ATC ATC CCT AGA TAA GCG -3'<br>3'- CTG GTC GAA TAG TAG GGA TCT ATT CGC -5' Matched DNA<br>3'- CTG GTC GAA TAG TAC GGA TCT ATT CGC -5' CC Mismatched DNA<br><br>5'- GAC CAG CTT ATC ATX CCT AGA TAA GCG -3'<br>3'- CTG GTC GAA TAG TAY GGA TCT ATT CGC -5'<br><br>X Y<br>A _ AP_A<br>T _ AP_T<br>C _ AP_C<br>G _ AP_G

Emission quenching experiment	5'- GAC CAG CTT ATC ATC CCT AGA TAA GCG -3'<br>3'- CTG GTC GAA TAG TAG GGA TCT ATT CGC -5' Matched DNA<br>3'- CTG GTC GAA TAG TAC GGA TCT ATT CGC -5' CC Mismatched DNA

Supplementary Table 2. Sequence and structures of nucleic acids used in this study.

Supplementary Methods

The DNA sequences are listed in the following table and they were purchased from Integrated DNA Technologies (IDT) of Centre for Genomic Sciences in The University of Hong Kong. To prepare DNA duplexes, complementary oligomers were mixed in 1:1 molar ratios, heated to 94 ℃, and slowly cooled to ambient temperature. ^{1}H NMR spectra were recorded on a Bruker DPX-300 or 400 FT-NMR spectrometer. Positive-ion mass spectra were recorded on a Finnigan MAT95 mass spectrometer. Elemental analyses were performed by the Institute of Chemistry at the Chinese Academy of Sciences, Beijing. HCT116 and HCT116N cell lines were maintained in dulbecco's modified eagle medium F12; prostate cancer cell line DU145 was cultured in eagle's minimum essential medium (MEM); colon cancer SW480 was maintained in dulbecco's modified eagle medium (DMEM) and NCM460 was cultured in Roswell Park Memorial Institute (RPMI) 1640 medium. All the culture media were supplemented with 10% fetal bovine serum, 100 U mL^{-1} penicillin, and 100 μg mL^{-1} streptomycin at 37 °C in humidified atmosphere with 5% CO_2. The structures of all nucleic acids are listed in Supplementary Table 2.

Experimental procedure and compound characterization

A) Synthesis and characterization of complexes

Synthesis of 1a

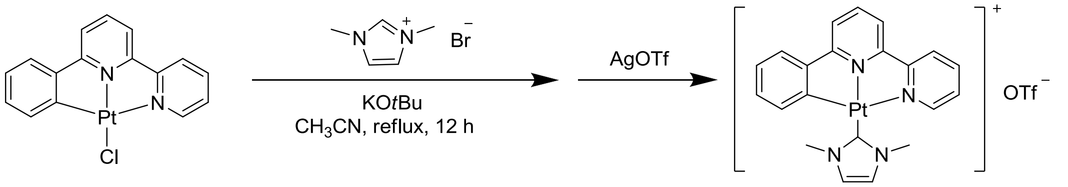

A mixture of [Pt(CNN)Cl] (50 mg, 0.108 mmol), potassium *tert*-butoxide (12 mg, 0.108 mmol) and 1,3-dimethyl-1H-imidazol-3-ium bromide (21 mg, 0.118 mmol) in acetonitrile (10 mL) was heated to reflux for 12 h. After cooling to room temperature, excess silver trifluoromethanesulfonate (84 mg, 0.33 mmol) was added into the reaction mixture and stirred for 30 min. After extracting the crude product into dichloromethane layer, it was purified by column chromatography on silica gel with CH_3CN/CH_2Cl_2 as eluent, and yellow powder was obtained.

Yield 58%; ^{1}H NMR (400 MHz, CD_3CN): δ = 8.29-8.19 (m, 3H), 8.11 (t, 1H, *J* = 8.0 Hz), 7.99 (d, 1H, *J* = 4.0 Hz), 7.88 (d, 1H, *J* = 4.0 Hz), 7.63-7.56 (m, 2H), 7.34-7.26 (m, 2H), 7.12 (t, 1H, *J* = 8.0 Hz), 7.05 (t, 1H, *J* = 8.0 Hz), 6.58-6.36 (m, 1H), 3.82 (s, 6H); ^{13}C NMR (100 MHz, CD_3CN): δ = 38.3, 120.2, 120.4, 120.7, 123.7, 125.2, 126.4, 129.5, 132.4, 137.6, 140.1, 141.6, 142.4, 148.6, 153.3, 155.3, 158.7, 164.1, 165.1; MS (FAB, +ve): *m/z* 522 $[M\text{-}OTf]^+$; Elemental analysis calcd (%) for $C_{22}H_{19}F_3N_4O_3PtS{\bullet}0.5CH_2Cl_2$: C 37.85, H 2.82, N 7.85; found: C 37.60, H 2.80, N 8.12.

Synthesis of 1b

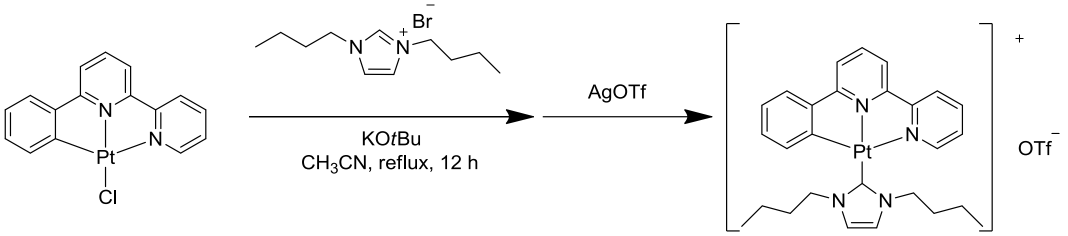

The procedure is similar to that for **1a**.

Yield 44%; ^{1}H NMR (400 MHz, CD_3CN): δ = 8.28-8.15 (m, 3H), 8.13-8.03 (m, 1H), 7.95 (d, 1H, *J* = 8.0 Hz), 7.84 (d, 1H, *J* = 8.0 Hz), 7.61-7.52 (m, 2H), 7.38-7.28 (m, 2H), 7.15-6.97 (m, 2H), 6.59-6.36 (m, 1H), 4.35-4.10 (m, 4H), 1.86-1.67 (m, 4H), 1.30-1.10 (m, 4H), 0.71 (t, 6H, *J* = 8.0 Hz); ^{13}C NMR (100 MHz, CD_3CN): δ = 13.7, 20.1, 33.2, 51.3, 120.4, 120.7, 122.6, 125.2, 125.8, 126.4, 129.5, 132.4, 137.8, 139.1, 140.6, 141.7, 142.5, 148.6, 153.1, 155.5, 159.0, 163.2; MS (FAB, +ve): *m/z* 606 $[M\text{-}OTf]^+$; Elemental analysis calcd (%) for $C_{28}H_{31}F_3N_4O_3PtS$: C 44.50, H 4.13, N

7.41; found: C 44.54, H 4.37, N 7.45.

Synthesis of 1c

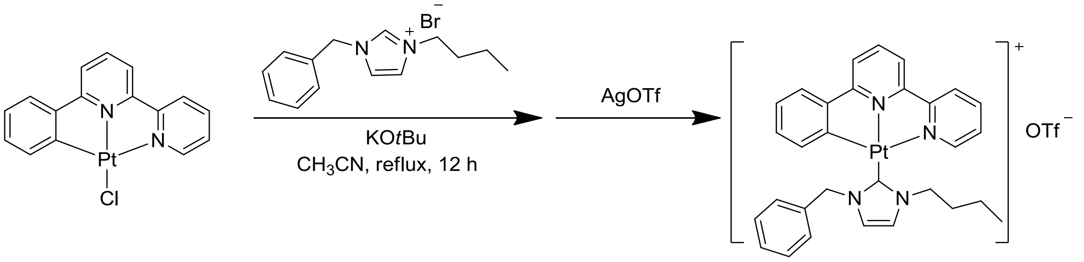

The procedure is similar to that for **1a**.

Yield 51%; ^{1}H NMR (400 MHz, CD_3CN): δ = 8.21-8.05 (m, 3H), 7.99-7.93 (m, 1H), 7.90-7.78 (m, 2H), 7.63-7.56 (m, 1H), 7.49-7.34 (m, 3H), 7.30-7.21 (m, 2H), 7.16-6.99 (m, 5H), 6.63-6.40 (m, 1H), 5.39 (s, 2H), 4.29-4.16 (m, 2H), 1.83-1.72 (m, 2H), 1.29-1.15 (m, 2H), 0.78-0.70 (t, 3H, *J* = 8.0 Hz); ^{13}C NMR (100 MHz, CD_3CN): δ = 13.7, 20.2, 33.1, 51.5, 55.2, 120.3, 120.6, 122.9, 123.1, 124.9, 125.8, 126.4, 128.9, 129.1, 129.3, 129.4, 132.5, 137.8, 139.1, 141.3, 142.5, 153.0; MS (FAB, +ve): *m/z* 640 $[M\text{-}OTf]^+$; Elemental analysis calcd (%) for $C_{31}H_{29}F_3N_4O_3PtS$: C 47.15, H 3.70, N 7.09; found: C 47.35, H 3.72, N 7.09.

Synthesis of 1d

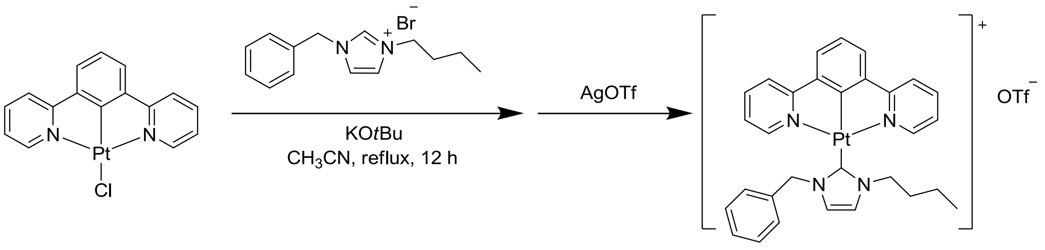

The procedure is similar to that for **1a**.

Yield 53%; ^{1}H NMR (400 MHz, CD_3CN): δ = 8.08-7.99 (m, 2H), 7.94-7.86 (m, 2H), 7.86-7.70 (m, 2H), 7.70-7.63 (m, 2H), 7.55-7.47 (m, 1H), 7.43-7.38 (m, 1H), 7.37-7.29 (m, 1H), 7.28-7.21 (m, 2H), 7.13-7.01 (m, 5H), 5.45-5.40 (m, 2H), 4.31-4.22 (m, 2H), 1.85-1.72 (m, 2H), 1.28-1.15 (m, 2H), 0.77-0.67 (m, 3H); ^{13}C NMR (125 MHz, CD_3CN): δ = 13.7, 20.1, 34.0, 51.1, 54.9, 121.5, 123.1, 123.7, 125.4, 125.8, 128.9, 129.0, 129.3, 137.9, 141.2, 144.0, 155.5, 170.0, 174.4, 188.8; MS (FAB, +ve): *m/z* 640 $[M\text{-}OTf]^+$; Elemental analysis calcd (%) for $C_{31}H_{29}F_3N_4O_3PtS{\bullet}0.5H_2O$: C 46.61, H 3.79, N 7.01; found: C 46.74, H 3.63, N 7.09.

Synthesis of 1e

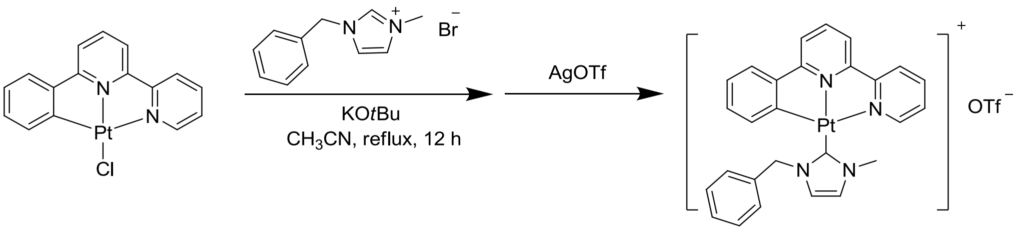

The procedure is similar to that for **1a**.

Yield 55%; ^{1}H NMR (400 MHz, CD_3CN): δ = 8.20-8.05 (m, 3H), 7.94 (d, 1H, *J* = 8.0 Hz), 7.85 (d, 2H, *J* = 8.0 Hz), 7.61-7.54 (m, 1H), 7.45-7.36 (m, 2H), 7.36-7.29 (m, 1H), 7.27-7.20 (m, 2H), 7.15-6.98 (m, 5H), 6.62-6.39 (m, 1H), 5.40 (s, 2H), 3.81 (s, 3H); ^{13}C NMR (100 MHz, CD_3CN): δ = 38.6, 55.1, 118.3, 120.3, 120.3, 120.6, 123.0, 124.0, 125.0, 125.8, 126.4, 128.9, 128.9, 128.9, 129.3, 129.4, 132.5, 137.7, 141.3, 142.4; MS (FAB, +ve): *m/z* 598 $[M\text{-}OTf]^+$; Elemental analysis calcd (%) for $C_{28}H_{23}F_3N_4O_3PtS{\bullet}H_2O$: C 43.92, H 3.29, N 7.32; found: C 43.90, H 2.96, N 7.37.

Synthesis of 1f

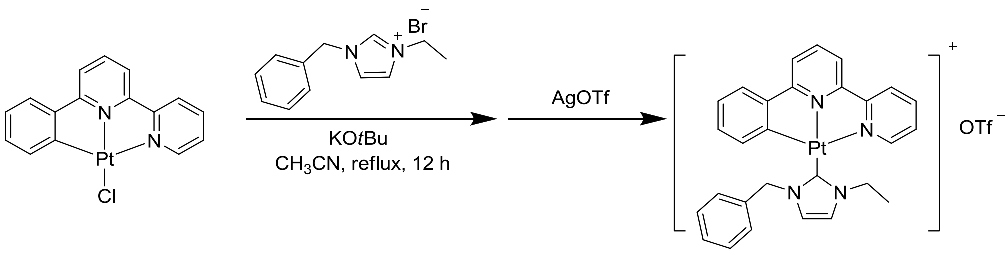

The procedure is similar to that for **1a**.

Yield 48%; ^{1}H NMR (400 MHz, $CDCl_3$): δ = 8.21-8.05 (m, 3H), 7.95 (d, 1H, *J* = 8.0 Hz), 7.89-7.75 (m, 2H), 7.62-7.55 (m, 1H), 7.45-7.37 (m, 3H), 7.29-7.22 (m, 2H), 7.15-7.01 (m, 5H), 6.61-6.40 (m, 1H), 5.40 (s, 2H), 4.30 (m, 2H), 1.34 (t, 3H, *J* = 8.0 Hz); ^{13}C NMR (100 MHz, $CDCl_3$): δ = 16.1, 46.4, 55.0, 119.3, 119.8, 121.2, 122.1, 124.7, 125.2, 125.4, 128.3, 128.5, 128.8, 131.8, 135.9, 136.9, 138.2, 139.4, 140.7, 141.6, 147.5, 151.8, 154.4, 157.5, 164.4; MS (FAB, +ve): *m/z* 612 $[M\text{-}OTf]^+$; Elemental analysis calcd (%) for $C_{29}H_{25}F_3N_4O_3PtS{\bullet}H_2O$: C 44.67, H 3.49, N 7.19; found: C 44.97, H 3.21, N 7.03.

Synthesis of 1g

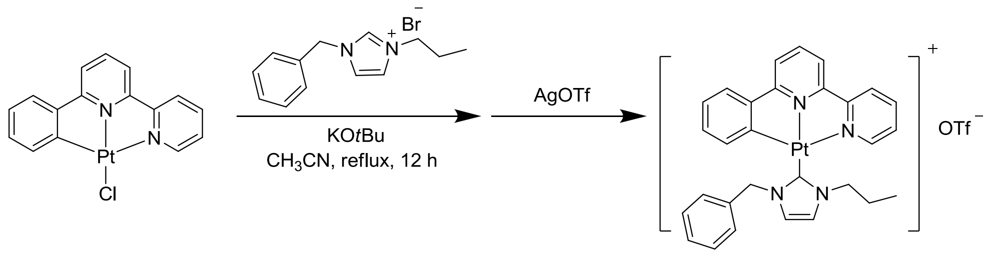

The procedure is similar to that for **1a**.

Yield 57%; ^{1}H NMR (400 MHz, CD_3CN): δ = 8.22-8.06 (m, 3H), 8.00-7.93 (m, 1H), 7.91-7.79 (m, 2H), 7.63-7.56 (m, 1H), 7.48-7.35 (m, 3H), 7.31-7.22 (m, 2H), 7.15-7.01 (m, 5H), 6.63-6.42 (m, 1H), 5.43 (s, 2H), 4.20 (t, 2H, *J* = 8.0 Hz), 1.85-1.80 (m, 2H), 0.83-0.79 (t, 3H, *J* = 8.0 Hz); ^{13}C NMR (100 MHz, CD_3CN): δ = 11.2, 24.5, 53.4, 55.2, 118.3, 120.3, 120.6, 122.9, 123.0, 124.9, 125.8, 126.4, 128.9, 129.3, 129.3, 132.4, 137.8, 141.3, 142.4, 152.9, 155.4; MS (FAB, +ve): *m/z* 626 $[M\text{-}OTf]^+$; Elemental analysis calcd (%) for $C_{30}H_{27}F_3N_4O_3PtS$: C 46.45, H 3.51, N 7.22; found: C 46.39, H 3.60, N 7.26.

Synthesis of 1h

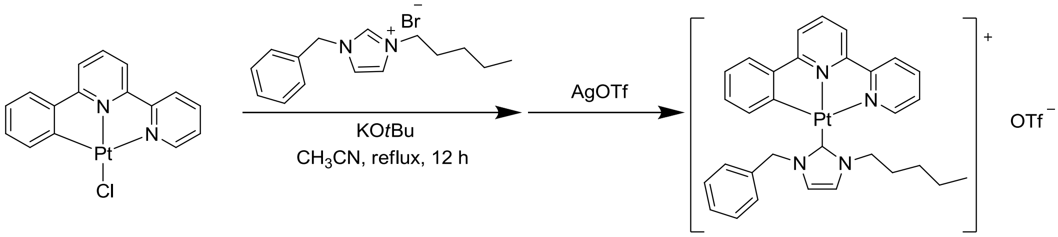

The procedure is similar to that for **1a**.

Yield 49%; ^{1}H NMR (300 MHz, CD_3CN): δ = 8.20-7.95 (m, 3H), 7.94-7.85 (m, 1H), 7.85-7.70 (m, 2H), 7.62-7.45 (m, 1H), 7.44-7.26 (m, 3H), 7.26-7.13 (m, 2H), 7.13-6.88 (m, 5H), 6.65-6.30 (m, 1H), 5.35 (s, 2H), 4.28-4.05 (m, 2H), 1.82-1.62 (m, 2H), 1.20-0.99 (m, 4H), 0.75-0.51 (m, 3H); ^{13}C NMR (100 MHz, CD_3CN): δ = 13.9, 22.7, 29.1, 30.7, 51.7, 55.2, 118.3, 120.3, 120.6, 122.9, 123.1, 124.9, 125.8, 126.4, 128.9, 129.3, 129.4, 132.4, 137.8, 139.1, 141.3, 142.5, 153.1; MS (FAB, +ve): *m/z* 654 $[M\text{-}OTf]^+$; Elemental analysis calcd (%) for $C_{32}H_{31}F_3N_4O_3PtS{\bullet}0.5CHCl_3$: C 45.21, H 3.68, N 6.49; found: C 45.03, H 3.57, N 6.53.

Synthesis of 1i

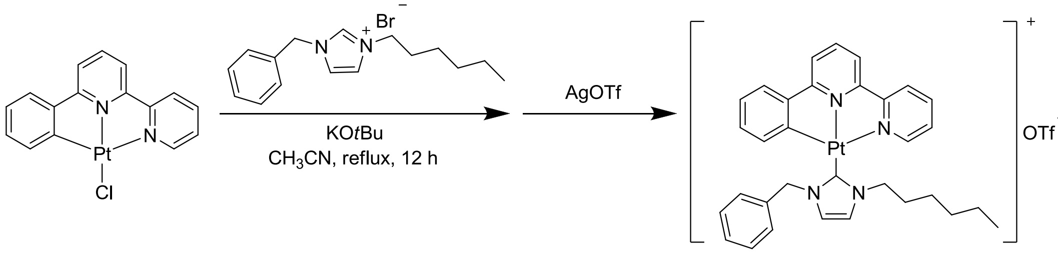

The procedure is similar to that for **1a**.

Yield 55%; ^{1}H NMR (400 MHz, CD_3CN): δ = 8.22-8.02 (m, 3H), 7.98-7.92 (m, 1H), 7.88-7.76 (m, 2H), 7.62-7.53 (m, 1H), 7.43-7.32 (m, 3H), 7.28-7.21 (m, 2H), 7.15-6.99 (m, 5H), 6.62-6.42 (m, 1H), 5.41-5.40 (m, 2H), 4.22 (t, 2H, *J* = 8.0 Hz), 1.88-1.75 (m, 2H), 1.20-0.98 (m, 6H), 0.65 (t, 3H, *J* = 12.0 Hz); ^{13}C NMR (100 MHz,

CD_3CN): δ = 14.0, 22.9, 26.5, 30.9, 31.7, 51.7, 55.2, 118.3, 120.3, 120.6, 122.9. 123.1, 125.0, 125.8, 126.4, 128.9, 129.4, 132.4, 137.8, 139.1, 141.3, 142.4, 153.0; MS (FAB, +ve): *m/z* 668 $[M-OTf]^+$; Elemental analysis calcd (%) for $C_{33}H_{33}F_3N_4O_3PtS{\bullet}0.5H_2O$: C 47.94, H 4.14, N 6.78; found: C 47.92, H 4.06, N 6.73.

Synthesis of 1j

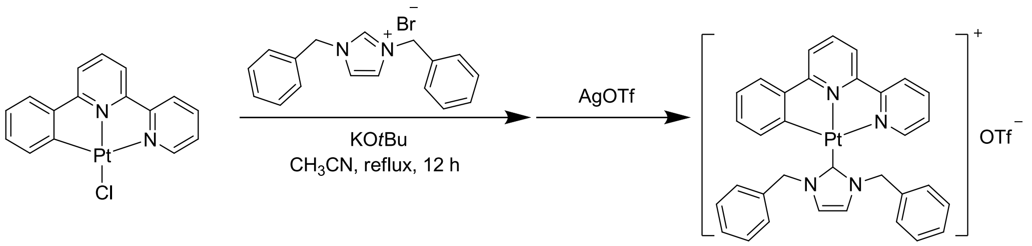

The procedure is similar to that for **1a**.

Yield 60%; ^1H NMR (400 MHz, CD_3CN): δ = 8.14-8.02 (m, 3H), 7.93 (d, 1H, *J* = 8.0 Hz), 7.85 (d, 1H, *J* = 8.0 Hz), 7.60 (d, 1H, *J* = 8.0 Hz), 7.45 (s, 2H), 7.43-7.33 (m, 1H), 7.30-7.19 (m, 5H), 7.18-7.01 (m, 8H), 6.67-6.45 (m, 1H), 5.45-5.30 (m, 4H); ^{13}C NMR (100 MHz, CD_3CN): δ = 55.4, 120.2, 120.5, 123.1, 123.3, 124.7, 125.8, 126.4, 129.0, 129.1, 129.4, 132.4, 137.6, 137.8, 140.3, 140.9, 142.4, 152.9, 158.1, 165.1; MS (FAB, +ve): *m/z* 674 $[M-OTf]^+$; Elemental analysis calcd (%) for $C_{34}H_{27}F_3N_4O_3PtS{\bullet}0.5H_2O$: C 49.04, H 3.39, N 6.73; found: C 49.03, H 3.28, N 6.77.

Synthesis of 1k

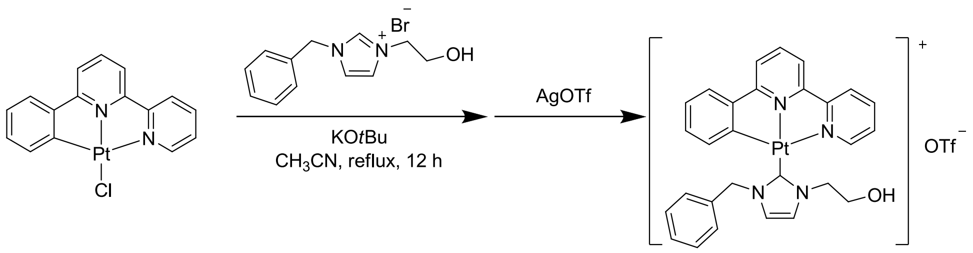

The procedure is similar to that for **1a**.

Yield 63%; ^1H NMR (400 MHz, $CDCl_3$): δ = 8.18-8.03 (m, 3H), 7.95 (d, 1H, *J* = 8.0 Hz), 7.89-7.78 (m, 2H), 7.64-7.55 (m, 1H), 7.48-7.36 (m, 3H), 7.30-7.22 (m, 2H), 7.16-7.02 (m, 5H), 6.62-6.42 (m, 1H), 5.44-5.41(m, 2H), 4.45-4.35 (m, 1H), 4.28-4.15 (m, 1H), 3.82-3.71(m, 2H); ^{13}C NMR (100 MHz, $CDCl_3$): δ = 53.8, 54.8, 61.3, 118.7, 119.1, 121.6, 123.1, 123.5, 125.1, 125.3, 128.2, 128.4, 128.8, 129.2, 132.0, 136.2, 136.8, 139.2, 140.0, 141.1, 147.7, 153.6, 154.3, 157.0, 163.2, 164.9; MS (FAB, +ve): *m/z* 628 $[M-OTf]^+$; Elemental analysis calcd (%) for $C_{29}H_{25}F_3N_4O_4PtS{\bullet}0.5H_2O$: C 44.28, H 3.33, N 7.12; found: C 44.10, H 3.12, N 7.18.

Synthesis of 1l

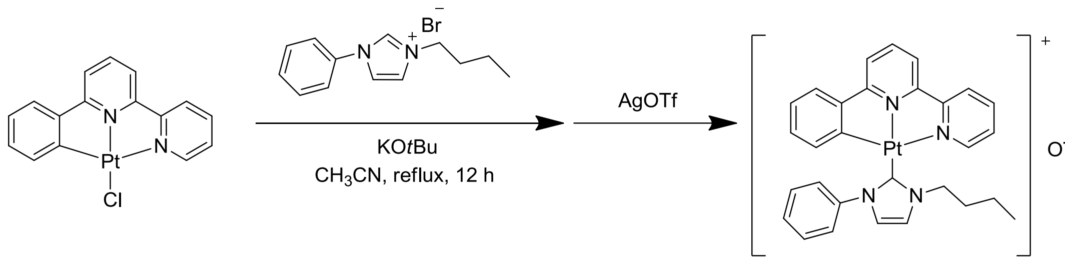

The procedure is similar to that for **1a**.

Yield 61%; ^{1}H NMR (400 MHz, $CDCl_3$): δ = 8.35-8.23 (m, 1H), 8.21-8.15 (m, 2H), 8.10-8.02 (m, 1H), 7.95-7.88 (m, 1H), 7.84-7.75 (m, 3H), 7.63-7.57 (m, 1H), 7.57-7.49 (m, 3H), 7.37-7.27 (m, 3H), 7.12-7.05 (m, 1H), 7.05-6.97 (m, 1H), 6.68-6.44 (m, 1H), 4.45-4.25 (m, 2H), 1.89-1.79 (m, 2H), 1.32-1.17 (m, 2H), 0.79-0.71 (m, 3H); ^{13}C NMR (100 MHz, $CDCl_3$): δ = 13.7, 19.8, 32.5, 51.5, 119.3, 119.8, 122.4, 122.8, 124.9, 125.1, 125.2, 125.3, 128.9, 129.6, 131.9, 137.0, 139.5, 139.6, 141.0, 141.5, 151.7, 154.6, 157.2, 162.4, 164.4; MS (FAB, +ve): *m/z* 626 $[M\text{-}OTf]^+$; Elemental analysis calcd (%) for $C_{30}H_{27}F_3N_4O_3PtS{\bullet}H_2O$: C 45.40, H 3.68, N 7.06; found: C 45.59, H 3.52, N 7.04.

Synthesis of 1m

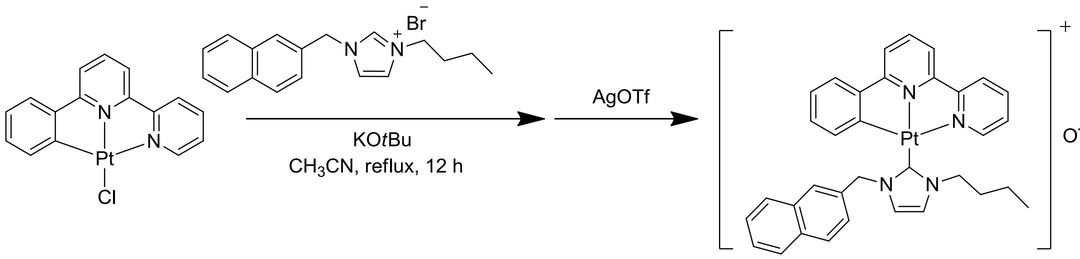

The procedure is similar to that for **1a**.

Yield 61%; ^{1}H NMR (400 MHz, CD_3CN): δ = 8.11-8.01 (m, 1H), 7.91-7.72 (m, 5H), 7.64-7.52 (m, 4H), 7.48-7.24 (m, 6H), 7.18-6.99 (m, 3H), 6.67-6.42 (m, 1H), 5.53-5.42 (m, 1H), 5.40-5.29 (m, 1H), 4.26-4.12 (m, 2H), 1.82-1.69 (m, 2H), 1.29-1.15 (m, 2H), 0.78-0.66 (m, 3H); ^{13}C NMR (100 MHz, CD_3CN): δ = 13.7, 20.2, 33.0, 51.8, 55.7, 118.3, 120.2, 120.5, 122.4, 123.7, 124.3, 125.7, 126.4, 126.7, 127.1, 127.3, 128.2, 128.4, 128.6, 129.2, 132.4, 133.3, 133.5, 135.4, 137.8, 140.5, 142.2, 152.2, 165.0; MS (FAB, +ve): *m/z* 690 $[M\text{-}OTf]^+$; Elemental analysis calcd (%) for $C_{35}H_{31}F_3N_4O_3PtS{\bullet}H_2O$: C 49.01, H 3.88, N 6.53; found: C 48.92, H 3.71, N 6.49.

Synthesis of 1n

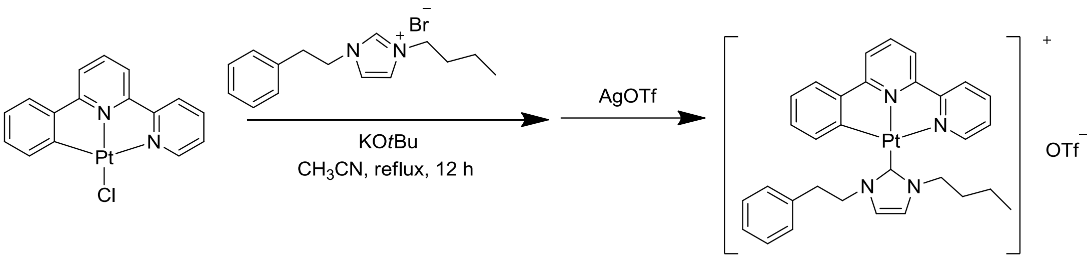

The procedure is similar to that for **1a**.

Yield 57%; ^{1}H NMR (400 MHz, CD_3CN): δ = 8.25-8.14 (m, 2H), 8.14-8.06 (m, 1H), 8.01-7.94 (m, 1H), 7.90-7.79 (m, 2H), 7.63-7.55 (m, 1H), 7.55-7.47 (m, 1H), 7.32-7.24 (m, 2H), 7.16-6.92 (m, 7H), 6.59-6.37 (m, 1H), 4.69-4.55 (m, 1H), 4.48-4.36 (m, 1H), 4.26-4.07 (m, 2H), 3.18-2.99 (m, 2H), 1.81-1.66 (m, 2H), 1.24-1.10 (m, 2H), 0.77-0.65 (m, 3H); ^{13}C NMR (100 MHz, CD_3CN): δ = 12.8, 19.2, 32.3, 36.3, 50.3, 52.1, 119.4, 119.7, 121.6, 121.7, 124.2, 124.9, 125.5, 126.7, 128.4, 128.7, 128.8, 131.6, 137.0, 137.9, 140.4, 141.5, 152.1; MS (FAB, +ve): *m/z* 654 $[M-OTf]^+$; Elemental analysis calcd (%) for $C_{32}H_{31}F_3N_4O_3PtS{\bullet}H_2O$: C 46.77, H 4.05, N 6.82; found: C 46.75, H 3.88, N 6.82.

Synthesis of 1o

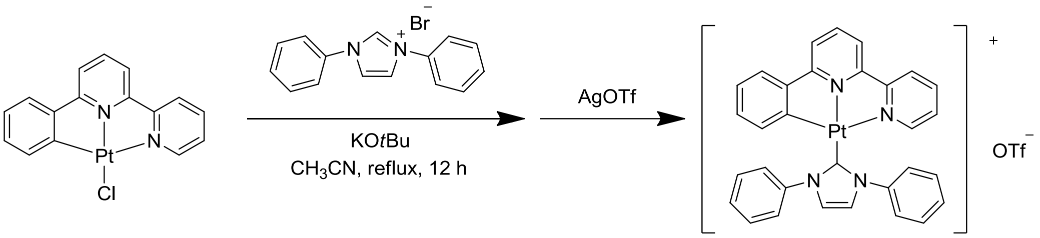

The procedure is similar to that for **1a**.

Yield 48%; ^{1}H NMR (400 MHz, $CDCl_3$): δ = 8.67-8.61 (m, 1H), 8.51-8.45 (m, 1H), 8.34-8.27 (m, 1H), 8.25-8.12 (m, 4H), 8.05-7.98 (m, 1H), 7.86-7.80 (m, 4H), 7.74-7.64 (m, 1H), 7.62-7.56 (m, 1H), 7.44-7.36 (m, 4H), 7.36-7.30 (m, 2H), 7.06-6.94 (m, 2H), 6.59-6.53 (m, 1H); ^{13}C NMR (100 MHz, CD_3CN): δ = 120.3, 120.6, 124.7, 125.2, 125.7, 126.3, 126.6, 129.6, 129.8, 130.2, 132.5, 137.7, 138.2, 140.2, 140.7, 141.6, 142.4, 153.5; MS (FAB, +ve): *m/z* 646 $[M-OTf]^+$; Elemental analysis calcd (%) for $C_{32}H_{23}F_3N_4O_3PtS{\bullet}0.5H_2O$: C 47.76, H 3.01, N 6.96; found: C 47.51, H 2.90, N 6.93.

Synthesis of 2

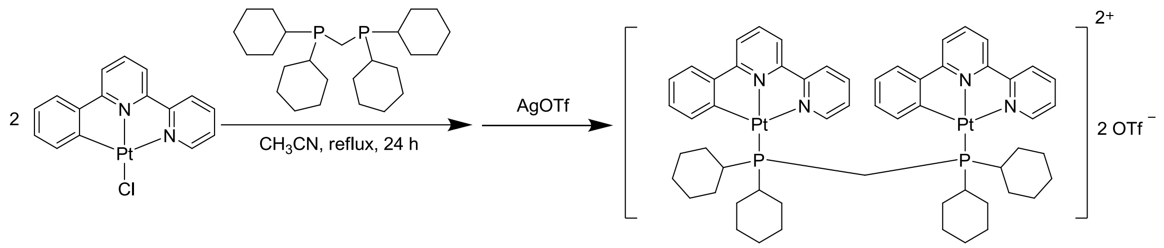

A mixture of [Pt(CNN)Cl] (50 mg, 0.108 mmol) and bis(dicyclohexylphosphino)methane (22 mg, 0.054 mmol) in acetonitrile (15 mL) was heated to reflux for 24 h. After cooling to room temperature, excess silver trifluoromethanesulfonate (84 mg, 0.33 mmol) was added into the reaction mixture and stirred for 30 min. After extracting the crude product into dichloromethane layer, it was purified by column chromatography on silica gel with CH_3CN/CH_2Cl_2 as eluent, and reddish orange powder was obtained.

Yield 41%; ^{1}H NMR (400 MHz, CD_3CN): δ = 8.06-7.82 (m, 4H), 7.82-7.71 (m, 4H), 7.71-7.55 (m, 4H), 7.48-7.11 (m, 8H), 7.03-6.88 (m, 2H), 5.44 (s, 2H), 3.36-0.65 (m, 44H); ^{13}C NMR (100 MHz, CD_3CN): δ = 15.6, 26.5, 27.5, 28.5, 30.8, 32.0, 34.3, 66.2, 120.8, 121.1, 124.8, 127.6, 127.8, 128.0, 132.2, 132.9, 141.7, 143.1, 148.0, 153.6, 153.9, 158.0, 163.4; MS (FAB, +ve): *m/z* 1261 $[M\text{-}2OTf+1]^+$; Elemental analysis calcd (%) for $C_{59}H_{68}F_6N_4O_6P_2Pt_2S_2$: C 45.44, H 4.40, N 3.59; found: C 45.39, H 4.53, N 3.40.

Synthesis of 2a

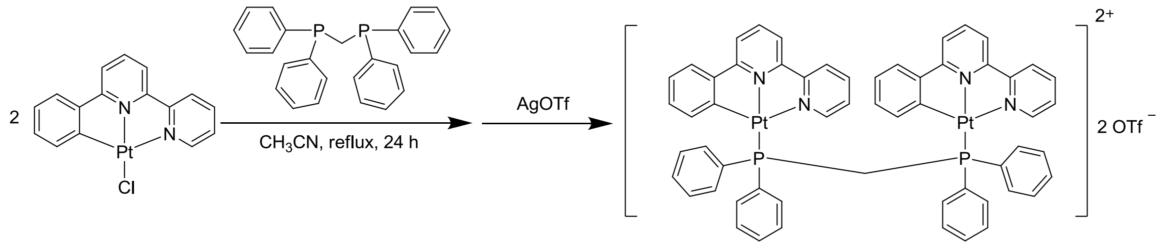

The procedure is similar to that for **2**.
Yield 41%; ^{1}H NMR (400 MHz, CD_3CN): δ = 8.40-8.02 (m, 4H), 7.90-7.88 (m, 4H), 7.88-7.75 (m, 6H), 7.68-7.60 (m, 2H), 7.52-7.31 (m, 14H), 7.00-6.88 (m, 2H), 6.78-6.60 (m, 4H), 6.52-6.35 (m, 4H), 6.25-6.10 (m, 2H), 4.95-4.71 (m, 2H); ^{13}C NMR (100 MHz, CD_3CN): δ = 120.7, 121.0, 124.9, 127.1, 127.3, 127.8, 130.1, 130.2, 131.5, 133.0, 139.1, 140.9, 143.6, 147.7, 152.1, 154.1, 157.4, 163.5; MS (FAB, +ve): *m/z* 1237 $[M\text{-}2OTf+1]^+$; Elemental analysis calcd (%) for

$C_{59}H_{44}F_6N_4O_6P_2Pt_2S_2$•$CH_2Cl_2$: C 44.48, H 2.86, N 3.46; found: C 44.68, H 2.94, N 3.63.

Synthesis of 3

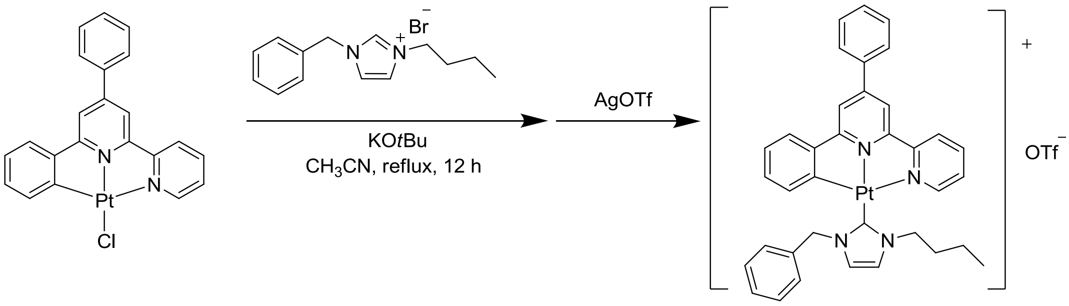

The procedure is similar to that for **1a**.

Yield 53%; ^{1}H NMR (400 MHz, CD_3CN): δ = 8.36-8.30 (m, 1H), 8.24-8.09 (m, 3H), 7.98-7.91 (m, 2H), 7.91-7.82 (m, 1H), 7.77-7.71 (m, 1H), 7.66-7.56 (m, 3H), 7.48-7.35 (m, 3H), 7.30-7.23 (m, 2H), 7.19-7.02 (m, 5H), 6.66-6.44 (m, 1H), 5.43 (s, 2H), 4.30-4.22 (m, 2H), 1.88-1.76 (m, 2H), 1.32-1.17 (m, 2H), 0.81-0.71 (m, 3H); ^{13}C NMR (100 MHz, CD_3CN): δ = 13.8, 20.2, 33.1, 51.5, 55.2, 122.9, 123.0, 125.1, 125.7, 126.5, 128.5, 128.9, 129.0, 129.3, 129.4, 130.2, 131.5, 132.4, 137.7, 137.8, 140.4, 141.2, 148.6, 153.0, 154.5, 155.6, 158.5, 163.8, 165.1; MS (FAB, +ve): *m/z* 716 $[M\text{-}OTf]^+$; Elemental analysis calcd (%) for $C_{37}H_{33}F_3N_4O_3PtS$: C 51.33, H 3.84, N 6.47; found: C 51.20, H 3.88, N 6.52.

Synthesis of 4

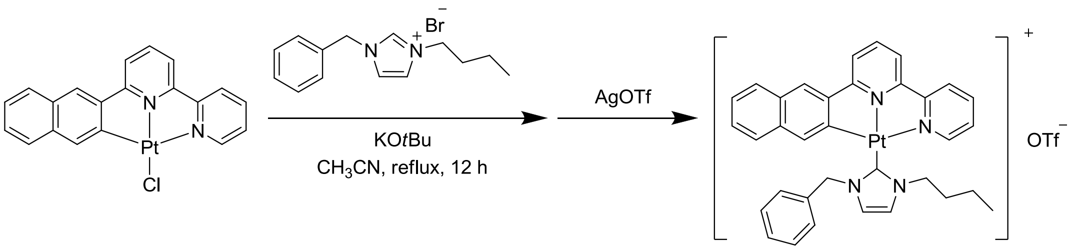

The procedure is similar to that for **1a**.

Yield 53%; ^{1}H NMR (400 MHz, $CDCl_3$): δ = 8.27-8.10 (m, 5H), 8.08-8.02 (m, 1H), 7.91-7.81 (m, 2H), 7.59-7.53 (m, 1H), 7.51-7.37 (m, 5H), 7.31-7.24 (m, 2H), 7.11-7.00 (m, 3H), 6.96-6.90 (m, 1H), 5.47 (s, 2H), 4.28 (t, 2H, J = 8.0 Hz), 1.90-1.72 (m, 2H), 1.30-1.14 (m, 2H), 0.71 (t, 3H, J = 8.0 Hz); ^{13}C NMR (100 MHz, CD_3CN): δ = 13.7, 20.1, 33.0, 51.5, 55.2, 121.1, 121.5, 123.0, 123.1, 124.9, 126.3, 126,4, 127.6, 128.7, 128.8, 128.9, 129.2, 129.3, 129.8, 132.2, 134.3, 136.1, 136.6, 137.8, 141.0, 141.9, 147.2, 152.8; MS (FAB, +ve): *m/z* 690 $[M\text{-}OTf]^+$; Elemental analysis calcd (%) for $C_{35}H_{31}F_3N_4O_3PtS$•$0.5H_2O$: C 49.53, H 3.80, N 6.60; found: C 49.71, H 3.71, N

6.52.

Synthesis of 5a

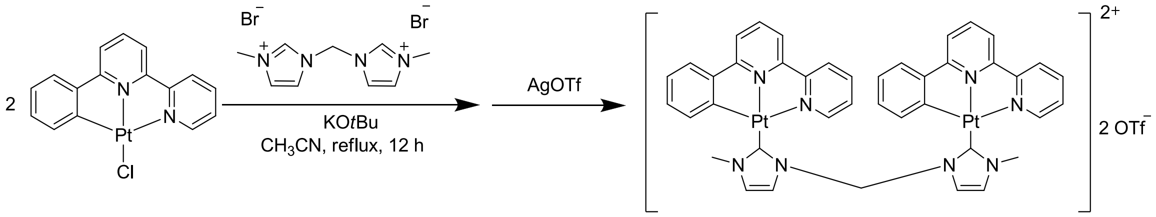

A mixture of [Pt(CNN)Cl] (50 mg, 0.108 mmol), potassium *tert*-butoxide (12 mg, 0.108 mmol), and 1,1′-methylenebis(3-methyl-1H-imidazol-3-ium) bromide (18.3 mg, 0.054 mmol) in acetonitrile (15 mL) was heated to reflux for 12 h. After cooling to room temperature, excess silver trifluoromethanesulfonate (84 mg, 0.33 mmol) was added into the reaction mixture and stirred for 30 min. After extracting the crude product into dichloromethane layer, it was purified by column chromatography on silica gel with CH_3CN/CH_2Cl_2 as eluent, and yellow powder was obtained.
Yield 58%; ^{1}H NMR (400 MHz, CD_3CN): δ = 8.02-7.90 (m, 4H), 7.87-7.78 (m, 6H), 7.58 (d, 2H, J = 8.0 Hz), 7.45 (d, 2H, J = 8.0 Hz), 7.42-7.36 (m, 2H), 7.16-7.08 (m, 4H), 6.92-6.81 (m, 4H), 6.71-6.62 (m, 2H), 6.41-6.18 (m, 2H), 3.55 (s, 6H); ^{13}C NMR (100 MHz, CD_3CN): δ = 38.8, 120.4, 120.5, 122.2, 125.3, 125.7, 125.8, 126.7, 128.9, 132.1, 137.0, 141.2, 142.3, 152.4; MS (FAB, +ve): *m/z* 1028 $[M-2OTf+1]^+$; Elemental analysis calcd (%) for $C_{43}H_{34}F_6N_8O_6Pt_2S_2$: C 38.92, H 2.58, N 8.44; found: C 38.75, H 2.61, N 8.44.

Synthesis of 5b

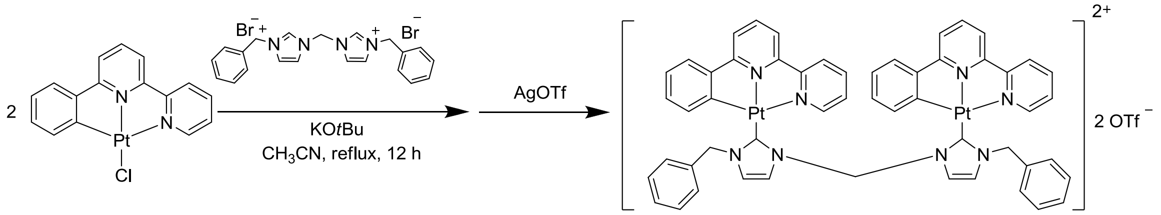

The procedure is similar to that for **5a**.
Yield 47%; ^{1}H NMR (400 MHz, CD_3CN): δ = 8.02-7.88 (m, 6H), 7.87-7.78 (m, 4H), 7.62-7.55 (m, 2H), 7.48-7.39 (m, 4H), 7.15-7.08 (m, 4H), 7.08-7.02 (m, 10H), 6.95-6.89 (m, 2H), 6.89-6.82 (m, 2H), 6.73-6.64 (m, 2H), 6.42-6.36 (m, 2H), 5.32-5.12 (m, 4H); ^{13}C NMR (100 MHz, CD_3CN): δ = 55.2, 120.5, 120.6, 123.1, 124.7, 125.3, 125.8, 126.7, 128.4, 129.0, 129.4, 132.2, 136.9, 137.3, 141.2, 142.4,

152.3; MS (FAB, +ve): *m/z* 1178 [M-2OTf+1]$^+$; Elemental analysis calcd (%) for $C_{55}H_{42}F_6N_8O_6Pt_2S_2$: C 44.66, H 2.86, N 7.58; found: C 44.51, H 2.67, N 7.44.

Synthesis of 5c

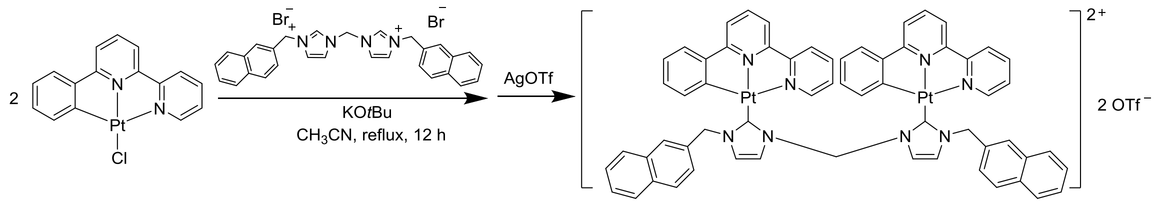

The procedure is similar to that for **5a**.
Yield 37%; ^{1}H NMR (400 MHz, CD_3CN): δ = 7.95-7.80 (m, 6H), 7.75-7.55 (m, 6H), 7.55-7.36 (m, 10H), 7.36-7.22 (m, 6H), 7.18-7.12 (m, 2H), 7.11-7.03 (m, 2H), 7.02-6.95 (m, 2H), 6.91-6.82 (m, 4H), 6.76-6.68 (m, 2H), 6.58-6.29 (m, 2H), 5.48-5.38 (m, 2H), 5.28-5.19 (m, 2H); ^{13}C NMR (100 MHz, CD_3CN): δ = 55.5, 120.3, 120.4, 122.8, 124.9, 125.1, 125.6, 126.0, 126.7, 127.2, 127.4, 127.9, 128.1, 128.3, 128.7, 129.1, 132.1, 136.9, 140.8, 142.1, 152.1; MS (FAB, +ve): *m/z* 1278 [M-2OTf+1]$^+$; Elemental analysis calcd (%) for $C_{63}H_{46}F_6N_8O_6Pt_2S_2$: C 47.91, H 2.94, N 7.09; found: C 47.66, H 3.01, N 7.22.

B) Characterization of binding with mismatched DNA

i) X-ray crystallographic analysis

Program(s) used to solve structure: SHELXS97 (Sheldrick, 1990); program(s) used to refine structure: SHELXL97 (Sheldrick, 1997)[1]. All esds (except the esd in the dihedral angle between two l.s. planes) are estimated using the full covariance matrix. The cell esds are taken into account individually in the estimation of esds in distances, angles and torsion angles; correlations between esds in cell structure report parameters are only used when they are defined by crystal symmetry. An approximate (isotropic) treatment of cell esds is used for estimating esds involving l.s. planes. Refinement of F^2 against ALL reflections. The weighted R-factor wR and goodness of fit S are based on F^2, conventional R-factors R are based on F, with F set to zero for negative F^2. The threshold expression of $F^2 > 2sigma(F^2)$ is used only for calculating R-factors(gt) etc. and is not relevant to the choice of reflections for refinement. R-factors based on F are statistically about twice as large as those based on F, and R-factors based on ALL data will be even larger. Satisfactory disorder models for the solvent and another trifilic counteranion of **2** were not found, and therefore the OLEX2 Solvent Mask routine (similar to PLATON/SQUEEZE) was used to mask out the disordered density.

ii) Emission titration experiment

Emission spectra were recorded on a SPEX Fluorolog-3 Model fluorescence spectrophotometer. The emission spectrum of a solution of different complexes (5 μM) in buffer solution (50 mM NaCl, 2 mM Tris, pH 7.5) was recorded and the solution was added with the aliquots of stock solution of different types of DNA (1 mM) and the emission spectra were recorded after equilibration for 3 min per aliquot until saturation point was almost reached. Nanosecond time-resolved emission measurements were performed on a LP920 Laser Flash Photolysis Spectrometer (Edinburgh Instruments Ltd., Livingston, UK). The excitation source was the 355 nm output (third harmonic) of a Nd:YAG laser (Spectra-Physics Quanta-Ray Lab-130 Pulsed Nd:YAG Laser). The signals were processed by a PC plug-in controller with L900 software.

iii) UV-visible absorption titration experiment

Absorption spectra were recorded on a Perkin-Elmer Lambda 900 UV-visible spectrophotometer. Absorption titration experiment was performed according to a reported procedure[2]. The absorption spectrum of a solution of **1c** (20 μM) in Tris buffer (50 mM NaCl, 2 mM Tris, pH 7.5) was recorded and the solution was added with the aliquots of stock solution of CC mismatched or matched DNA (1 mM) and the absorption spectra were recorded after equilibration for 1 min per aliquot.

iv) Isothermal titration calorimetry (ITC) experiment

ITC measures the heat absorbed or generated during a bi-molecular binding event[3]. To determine the binding constant of Pt(II) complexes with CC mismatched DNA and matched DNA, 0.1 mM of Pt(II) complex in an aqueous buffer solution (50 mM NaCl, 2 mM Tris, pH 7.5) was titrated by 0.75 mM of DNA. Heat released from each step of titration had been recorded.

Through accurate measurement of the heat changes in each step of the titration and fitting the data by using a non-liner least square method, the best-fitted graph would yield the binding constant (K) of the bi-molecular interaction.

v) NMR titration experiment

The experiments were performed with a Bruker DRX500 spectrometer at 283 K. Typical acquisition conditions for a ^{1}H NMR spectrum were 458 pulse length, 2.0 s relaxation delay (4 s for determination of formation constants), 16000 data points and 16±32 transients.

The palindromic DNA with sequence 5′- CGGACTCCG -3′ was dissolved in 50 mM sodium phosphate buffer at pH 6.10 containing 20 mM NaCl. The final concentration of duplex DNA was 1.3 mM. Then, the solvent was lyophilized from the aqueous buffer, then twice redissolved in 99.9% D_2O and lyophilized, and finally redissolved in 500 μL of 99.9% D_2O. The D_2O signal was used as an internal reference (δ = 4.80). Aliquots of stock solutions of **1c** (40 mM) were titrated directly to the DNA solution in an NMR tube. After mixing for 30 s, the NMR spectra were recorded. The 2D NMR parameters were set following a previous report[4].

vi) Molecular docking model

A model study on the binding mode between the Pt complex and the CC mismatched DNA was carried out. The complex structure optimizations were calculated by using Gaussian 09[5]. Their geometries were optimized *via* DFT calculations using the M06L functional[6]. Frequency calculation at the same level of theory has also been performed in order to identify all of the stationary points as minima. The 6-31G* basis set[7,8] was used for all atoms except for Pt, which was described by the Stuttgart small-core relativistic effective-core potential with its accompanying basis set[9]. The solvent effect was included by SCRF calculation based on the Polarizable Continuum Model (PCM)[10,11], in which water was used as the solvent. The optimized structures of Pt complexes were used to do the docking simulation. The docking process was performed by ICM-Pro 3.6-1d program (Molsoft)[12]. The energies were calculated with the all-atom vacuum force field ECEPP/3 in which solvation free energy and entropic contribution are considered by additional terms. The biased probability Monte Carlo (BPMC) procedure was employed on the conformational sampling which consists of 1) random move in the internal coordinate space followed by conformational change according to a predefined continuous probability distribution; 2) full local energy minimization with differentiable terms; 3) calculation of desolvation energy; 4) acceptance or rejection of the final minimized conformation based on Metropolis selection criterion and the whole procedure was repeated until the maximal number of steps is achieved[13]. The binding energy between the Pt complex and CC mismatched DNA includes grid potential energy, entropy loss and solvation energy differences. The crystal structures of Adenosine-Cytosine (AC) mismatched DNA (PDB code: 2O1I)[14] and Adenosine-Adenosine (AA) mismatched DNA (PDB code: 3GSK)[15] were downloaded from protein data bank, and the mismatched part was manually mutated into Cytosine-Cytosine (CC) pairs. These DNAs were our trial DNAs in the docking simulation. The docking process was performed to estimate the most possible binding modes and their corresponding binding energies. These binding modes between Pt complexes and CC mismatched DNA of matched and mismatched bindings were both successfully captured. The binding energies of **1a**, **1c** and **2** to mismatched DNA are -71.9, -84.8 and -129.8 kcal mol^{-1}, respectively, which are all higher than the corresponding binding energies of -68.3, -55.3 and -82.7 kcal mol^{-1} to matched DNA.

vii) Melting temperature experiment[4,16]

Melting experiments were conducted on a single beam Beckman DU 650 spectrophotometer with a Micro Tm Analysis accessory, High Performance Peltier controller. The absorption spectrum of a solution of CC mismatched DNA**,** matched or abasic DNA (2 μM) in buffer solution (50 mM NaCl, 2 mM Tris, pH 7.5) was

recorded. The absorbance values at 260 nm were monitored every 2 ℃ in the temperature range of 18-68 ℃. The graph of absorbance change at 260 nm against different temperature was plotted. Finally, the melting temperature could be obtained by finding the mid-point value of the sigma curve.

viii) Emission Quenching experiment[17]

Equal molar ratio of complex (**1c** or **2**) and 27-mer duplex CC mismatched DNA (5 μM) were mixed in buffer solution (50 mM NaCl, 2 mM Tris, pH 7.5). Then, different amounts of $[Cu(phen)_2]^{2+}$ (1:0, 1:1, 1:5, 1:10 and 1:100 of complex: $[Cu(phen)_2]^{2+}$) was added and the emission spectra were recorded after equilibration for 30 min.

Supplementary References

1. Sheldrick, G. M. A short history of SHELX. *Acta Cryst.* **A64,** 112-122 (2008).
2. McGhee, J. D. & von Hippel, P. H. Theoretical aspects of DNA-protein interactions: Co-operative and non-co-operative binding of large ligands to a one-dimensional homogeneous lattice. *J. Mol. Biol.* **86,** 469-489 (1974).
3. Ladbury, J. E. & Chowdhry, B. Z. Sensing the heat: the application of isothermal titration calorimetry to thermodynamic studies of biomolecular interactions. *Chem. Biol*. **3,** 791-801 (1996).
4. Cordier, C., Pierre, V. C. & Barton, J. K. Insertion of a bulky rhodium complex into a DNA cytosine-cytosine mismatch: an NMR solution study. *J. Am. Chem. Soc.* **129,** 12287-12295 (2007).
5. Frisch, M. J. *et al. Gaussian 09,* Wallingford CT, (2009).
6. Zhao, Y. & Truhlar, D. G. A new local density functional for main-group thermochemistry, transition metal bonding, thermochemical kinetics, and noncovalent interactions. *J. Chem. Phys*. **125,** 194101 (2006).
7. Hariharan, P. C. & Pople, J. A. The influence of polarization functions on molecular orbital hydrogenation energies. *Theoret. Chim. Acta* **28,** 213-222 (1973).
8. Francl, M. M., Pietro, W. J., Hehre, W. J., Binkley, J. S., Gordon, M. S., Defrees, D. J. & Pople, J. A. Self-consistent molecular orbital methods. XXIII. A polarization-type basis set for second-row elements. *J. Chem. Phys*. **77,** 3654-3665 (1982).
9. Andrae, D., Häußermann, U., Dolg, M., Stoll, H. & Preuß, H. Energy-adjusted *ab initio* pseudopotentials for the second and third row transition elements. *Theoret. Chim. Acta* **77,** 123-141 (1990).
10. Miertuš, S., Scrocco, E. & Tomasi, J. Electrostatic interaction of a solute with a continuum. A direct utilizaion of AB initio molecular potentials for the prevision of solvent effects. *Chem. Phys*. **55,** 117-129 (1981).
11. Tomasi, J., Mennucci, B. & Cammi, R. Quantum mechanical continuum solvation models. *Chem. Rev*. **105,** 2999-3094 (2005).
12. Totrov, M. & Abagyan, R. Flexible protein-ligand docking by global energy optimization in internal coordinates. *Proteins* **Suppl 1,** 215-220 (1997).
13. Neves, M. A. C., Totrov, M. & Abagyan, R. Docking and scoring with ICM: the benchmarking results and strategies for improvement. *J. Comput. Aided Mol. Des*. **26,** 675-686 (2012).
14. Pierre, V. C., Kaiser, J. T. & Barton, J. K. Insights into finding a mismatch through the structure of a mispaired DNA bound by a rhodium intercalator. *Proc. Natl.*

Acad. Sci. U.S.A. **104,** 429-434 (2007).
15. Zeglis, B. M., Pierre, V. C., Kaiser, J. T. & Barton, J. K. A bulky rhodium complex bound to an adenosine-adenosine DNA mismatch: General architecture of the metalloinsertion binding mode. *Biochemistry* **48,** 4247-4253 (2009).
16. Owczarzy, R., You, Y., Moreira, B. G., Manthey, J. A., Huang, L., Behlke, M. A. & Walder, J. A. Effects of sodium ions on DNA duplex oligomers: Improved predictions of melting temperatures. *Biochemistry* **43,** 3537-3554 (2004).
17. Lim, M. H., Song, H., Olmon, E. D., Dervan, E. E. & Barton, J. K. Sensitivity of $Ru(bpy)_2dppz^{2+}$ luminescence to DNA defects. *Inorg. Chem.* **48,** 5392-5397 (2009).
